# A non-toxic, reversibly released imaging probe for oral cancer that is derived from natural compounds

**DOI:** 10.1038/s41598-021-93408-0

**Published:** 2021-07-07

**Authors:** Magda Ghanim, Nicola Relitti, Gavin McManus, Stefania Butini, Andrea Cappelli, Giuseppe Campiani, K. H. Mok, Vincent P. Kelly

**Affiliations:** 1 School of Biochemistry and Immunology, Trinity Biomedical Sciences Institute, Trinity College Dublin, Dublin 2, Ireland; 2 Department of Biotechnology, Chemistry and Pharmacy, Department of Excellence 2018-2022, University of Siena, 53100 Siena, Italy

**Keywords:** Biochemistry, Cancer, Medical research, Oncology

## Abstract

CD44 is emerging as an important receptor biomarker for various cancers. Amongst these is oral cancer, where surgical resection remains an essential mode of treatment. Unfortunately, surgery is frequently associated with permanent disfigurement, malnutrition, and functional comorbidities due to the difficultly of tumour removal. Optical imaging agents that can guide tumour tissue identification represent an attractive approach to minimising the impact of surgery. Here, we report the synthesis of a water-soluble fluorescent probe, namely HA-FA-HEG-OE (compound **1**), that comprises components originating from natural sources: oleic acid, ferulic acid and hyaluronic acid. Compound **1** was found to be non-toxic, displayed aggregation induced emission and accumulated intracellularly in vesicles in SCC-9 oral squamous cells. The uptake of **1** was fully reversible over time. Internalization of compound **1** occurs through receptor mediated endocytosis; uniquely mediated through the CD44 receptor. Uptake is related to tumorigenic potential, with non-tumorigenic, dysplastic DOK cells and poorly tumorigenic MCF-7 cells showing only low intracellular levels and highlighting the critical role of endocytosis in cancer progression and metastasis. Together, the recognised importance of CD44 as a cancer stem cell marker in oral cancer, and the reversible, non-toxic nature of **1**, makes it a promising agent for real time intraoperative imaging.

## Introduction

Oral squamous cell carcinoma (OSCC) constitutes about 90% of malignancies of the oral cavity and its rate of incidence in young individuals has been rising in recent years^1–8^. The gold standard in oral cancer therapy—with the highest survival rates—continues to be surgical intervention^9^. However, tumours in the oral cavity often lack clearly demarcated edges, thereby adding to the difficulty of sparing adjacent healthy tissue during surgery. Broadening the resection margins can translate to permanent disfigurement, aesthetic and functional comorbidities, and a necessity for reconstructive surgery with consequences for the mental and physical wellbeing of the patient^10^. Therefore, the development of novel imaging modalities that can facilitate early tumour detection and that can be used intraoperatively to guide tumour resection are of clinical importance.

Non-specific fluorescent dyes, such as fluorescein isothiocyanate (FITC), indocyanine green (ICG) and 5-aminolevulinic acid (5-ALA) have been used in a clinical setting for many decades and across multiple tumour types to localize malignant cells during surgery^11^. However, these dyes are not target specific and their diagnostic sensitivity can be affected by their accumulation in normal or inflamed tissue, and furthermore, they have a propensity to interact non-specifically with plasma proteins, causing high background fluorescence, false positives, or photobleaching^12^. To address these problems, ongoing research efforts are exploiting fluorescently labelled antibodies, molecules, and peptides capable of interacting with cancer specific markers. For example, in oral cancer, fluorescently labelled antibodies that target PARP1 and EGFR have been shown to successfully image tumours pre- and intra-operatively; however, they have not yet gained clinical use^13, 14^.

Previously, Cappelli and colleagues designed several glycosaminoglycan hyaluronic acid (HA) macromolecules that are decorated with varying densities of ferulic acid (FA) residues that in turn, are linked to an oleic acid (OA) moiety *via* an amide bond^15–18^. Due to the presence of the fatty acid, the HA-FA containing copolymers are amphiphilic and can self-assemble to form aggregates in an aqueous environment. Interestingly, they also exhibited aggregation induced emission (AIE), a phenomenon observed with select organic fluorophores, wherein the compound shows significantly higher fluorescence emission upon generating uniformly distributed aggregates in solution^17^. It has been suggested that the presence of OA, could allow the polymers to interact with the lipid compartment of the cellular membrane. The internalization of the HA-FA copolymers to the cell cytoplasm was confirmed in NIH3T3 cells by fluorescent microscopy and in PANC-1 pancreatic cancer cells by confocal microscopy^17^.

**Figure 1:**
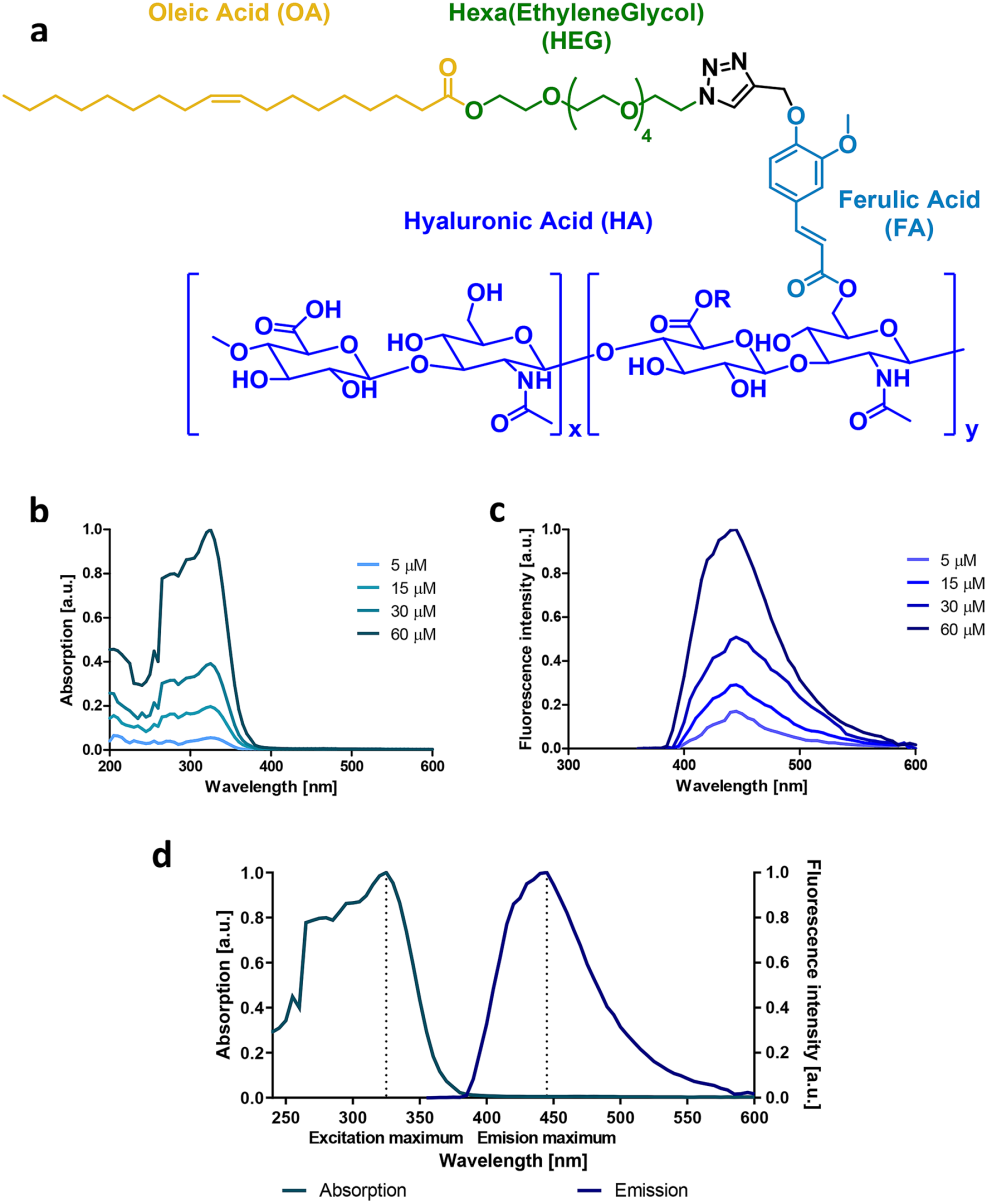
Chemical structure and optical properties of compound **1**. (**a**) Chemical structure of compound **1**. The modular structure of compound **1** comprises: hyaluronic acid (HA, navy blue), ferulic acid (FA, turquoise), glycol component (HEG, green) and OA (OA, yellow), 1,2,3-triazole (black). Ferulic acid has fluorescent properties induced by aggregation. Chemical structure drawn using ChemDraw version 4.5 software, PerkinElmer Informatics, www.informatics.perkinelmer.com. (**b**) Normalized absorption of compound **1** measured in MilliQ H_2_O at the indicated concentrations; (**c**) Normalized emission spectra of compound **1** at an excitation of 325 nm, measured in MilliQ H_2_O at the indicated concentrations; (**d**) Normalized absorption (green) and emission at 325 nm (blue) spectra of compound **1**, measured in MilliQ H_2_O at the concentration of 60 μM. a.u. arbitrary units. Graphs were plotted using GraphPad version 6.0c for Mac, www.graphpad.com.

In this study, based on the previous structure of our polymeric compound HA–FA–NEG–OA^17, 18^ we have created a new copolymer by the juxtaposition of different portions. In the new copolymer the amide bond bridging the ferulic acid and oleic acid moieties is replaced by an ester bond, and the nona(ethyleneglycol) (NEG) is replaced by a hexa(ethyleneglycol) (HEG) resulting in the molecule HA-FA-HEG-OE (**1**, Fig. 1a). We observe that compound **1** displays improved properties as an imaging agent, showing an increased fluorescence and Stokes shift relative to previous HA–FA–Pg molecules. In SCC-9 cells we show that **1** is internalised through the CD44 receptor into intracellular vesicles and does not gain access to the cell cytoplasm even after extended incubation times. The imaging agent is only weakly internalised to intracellular vesicles of non- and low-tumorigenic cells. Compound **1** shows no detrimental impact on cell viability or proliferation and can be readily released from cells upon dilution.

## Results

### Synthesis and fluorescent properties of Compound 1

Previously, we designed a set of synthetic HA-FA copolymers comprising an OA moiety bound through an amide glycol linker to a HA-FA backbone^17^. Here, we replaced the amide bond with an ester bond to provide greater structural flexibility, with the intention of improving its Stokes shift by the introduction of a polar bond (Fig. 1a)^19^.

Briefly, the target compound **1** (Fig. 1a) features four portions that were linked by the application of conventional in-solution chemistry procedures (Supplementary Fig. S1). Hexaethylene glycol (**2**) was mono-protected with *tert*-butyldimethylsilyl chloride (TBSCl) and imidazole affording compound **3**. The hydroxyl group of **3** was activated with mesyl chloride (MsCl) and then used in a nucleophilic substitution with sodium azide, thus generating compound **4**. This latter intermediate was deprotected with tetrabutylammonium fluoride (TBAF), and the corresponding alcohol was reacted with OA and 1,1’-carbonyldiimidazole (CDI) providing compound **5**. The HA–FA–Pg-3F (**6**) portion was synthesized as reported by Cappelli *et al.* in 2018^17^ and used in a click chemistry protocol with the azide **5** generating the desired product **1** (Supplementary Fig. S2-3). In a similar approach the triazole derivative **7** was isolated starting from **5** and methyl ferulate (Chemical synthesis described in detail in the Supplementary Information section).

As reported previously, FA and HA-FA containing molecules exhibit aggregation-induced emission (AIE) which could be exploited in the context of live tumour imaging to reduce background fluorescence^19^. Compound **1** shows good water solubility (mM range) and in aqueous solution displays an excitation maximum at 325 nm with a shoulder at 295 nm, suggesting that the absorbance spectrum of the polymer is dominated by the FA group (Fig. 1b). The emission maximum of **1** was detected at 445 nm (Fig. 1c), similar to values reported for FA alone in a solvent of high polarity^19^. The observed Stokes shift of 120 nm (Fig. 1d) was found to be equivalent to or better than previously published HA-FA polymers providing greater separation of the excitation and emission spectra^17^. This property grants higher precision in imaging through the avoidance of self-quenching.

### Compound 1 is reversibly internalised by SCC-9 cells

**Figure 2:**
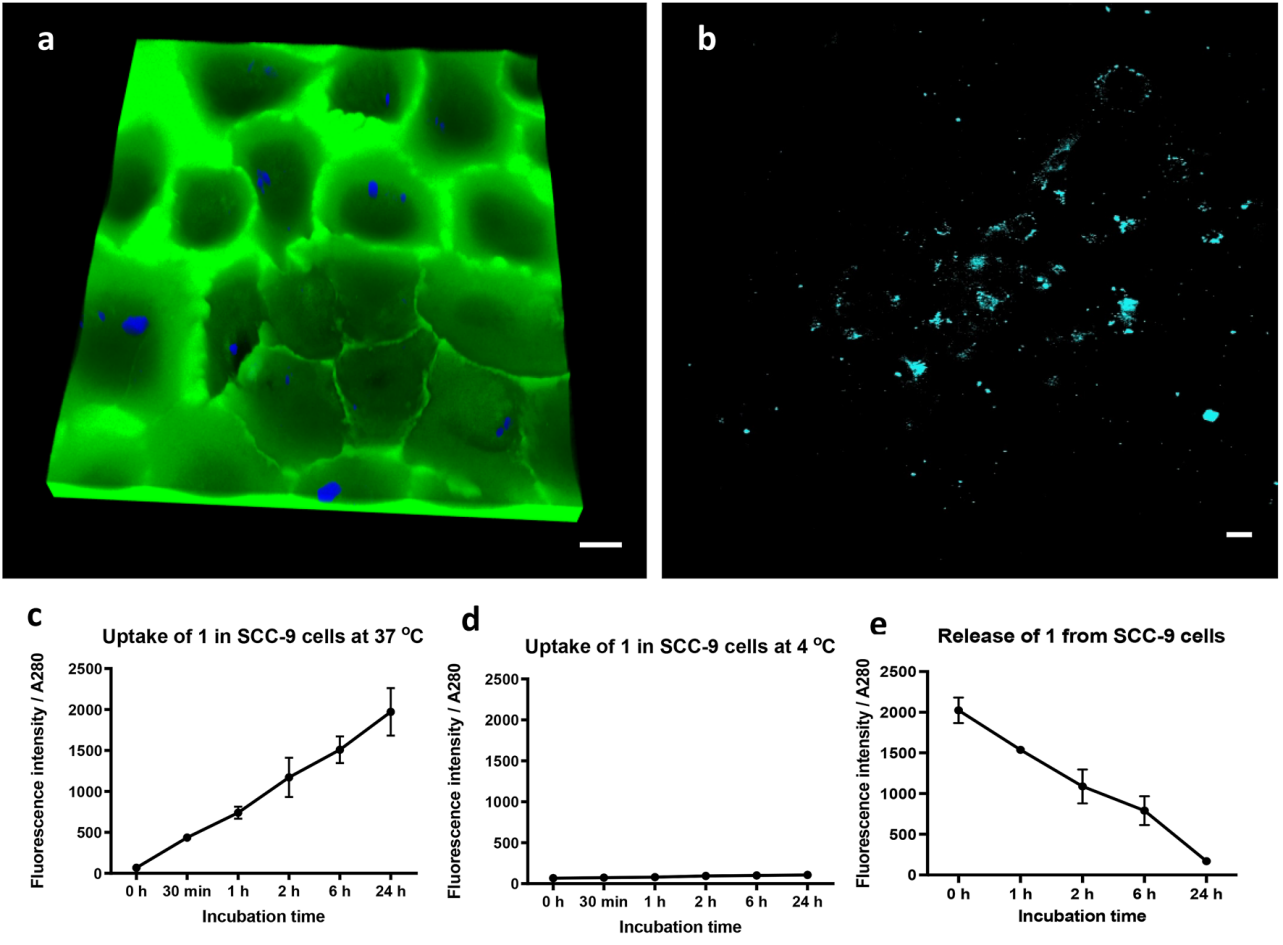
Internalization and release of compound **1** by SCC-9 cells. (**a**) 3D confocal microscopy of compound **1** in SCC-9 cells. Immediately prior to imaging, a concentration of 0.1 μM fluorescein sodium salt was added to the medium to allow visualisation of the cell outline. Bar is 20 μm. Images were analysed using Leica Application Suite X version 3.5.5.19976, www.leica-microsystems.com, 3D rendering was performed using Imaris version 9.5, www.imaris.oxinst.com. (**b**) Multiphoton microscopy of compound **1** in SCC-9 cells. Bar is 20 μm. Images were analysed using Olympus Fluoview FV10-ASW version 4.0, www.olympus-lifescience.com. (**c**,**d**) Analysis of time-dependent uptake of **1** by SCC-9 cells. Cells were treated with 5 μM **1** at c 37 °C, and d 4 °C before harvesting at the indicated time points. The fluorescence of the cell lysate was corrected against the absorbance at 280 nm. Means ± SD, n = 3. (**e**) Time-dependent release of **1** from SCC-9 cells. Cells were treated with 5 μM **1** for 24 h. Subsequently, medium was removed, cells were washed, and fresh medium added for the time intervals shown. The fluorescence of the cell lysate was taken and corrected against the absorbance at 280 nm. Means ± SD, n = 3. Graphs were plotted using GraphPad version 6.0c for Mac, www.graphpad.com.

Previously, HA-FA-copolymers were shown to localise into the cytoplasm of NIH3T3 and PANC-1 cells^17^. However, the uptake mechanism, subcellular localisation and expulsion of these molecules was not determined in detail. To examine the uptake of **1**, confocal studies were performed on SCC-9 cells—a tongue squamous cell carcinoma cell line. Cells were treated with **1** two hours prior to the addition of 0.01% fluorescein sodium salt to the medium, the latter allowing the outline of the cells and the extracellular space to be visualised through green fluorescence (Fig. 2a, Supplementary Video 1). Three-dimensional imaging showed that **1** accumulates within discrete regions in the cell interior (blue fluorescence) and that it is not dispersed throughout the cytoplasm. In accordance with our earlier observations, the accumulation of **1** in vesicles has the discrete advantage of aggregation-induced emission (AIE) thereby minimising background florescence.

Since the excitation maximum of **1** is within the ultraviolet range, this imaging approach would be unsuitable for live cell or tissue examination. However, the Stokes shift of 120 nm could be exploited in multiphoton imaging; a technique that exploits multiple lower energy photons to induce an excited fluorescent state^20^. The lower energy light used in multiphoton imaging is non-harmful and because of the longer wavelength, can penetrate deeper into tissues^21^. To test if **1** could be excited by longer wavelengths, multiphoton imaging was performed on SCC-9 cells (Fig. 2b). Notably, using an identical concentration and duration of **1** treatment as that of the confocal studies (20 μM, 2 h), the quantity of visualised molecule was much greater in multiphoton microscopy; an excitation wavelength of 730 nm and an emission wavelength range of 500-550 nm was experimentally determined to represent the highest signal-to-noise ratio. **1** was again observed to localise to discrete regions of the cell and did not disperse into the cytoplasm.

As the confocal microscopy analysis revealed a relatively efficient level of **1** internalisation, a time course was performed to examine the rate of uptake. Experiments were performed at both 37 °C and 4 °C, to distinguish between passive, energy-independent internalisation, and active transport. At 37 °C, with a concentration of 5 µM of compound, detectable levels of **1** fluorescence could be observed within 30 min (Fig. 2c). The uptake was found to remain linear up to 24 h post-treatment suggesting the process is not saturable. By contrast, in the lysates of cells that were maintained at 4 °C, only basal levels of fluorescence were observed (Fig. 2d), suggesting that only negligible amounts of **1** bound to the cell membrane and further, that **1** internalisation is energy dependent and, most probably, mediated through one or more endocytic pathways.

Since **1** was not observed to be present in the cytoplasm of SCC-9 cells, the release of the compound was examined over time. Cells were treated with **1** for 24 h, then washed and incubated in fresh medium for up to a further 24 h. A notable decrease in **1** fluorescence was observed in cellular lysates within 1 h of changing the medium, and a linear reduction in florescence was observed over time to almost undetectable levels by 24 h (Fig. 2e). The results corroborate the idea that **1** does not partition to the cell cytoplasm but instead is accumulated in cellular (endocytic) vesicles where it can be released following vesicle recycling. It was anticipated that the ready expulsion of the compound is indicative of a non-reactive, non-toxic agent that would be suitable for topical administration in oral cancer detection.

### Internalisation of Compound 1 by SSC-9 cells occurs via the CD44 receptor

**Figure 3:**
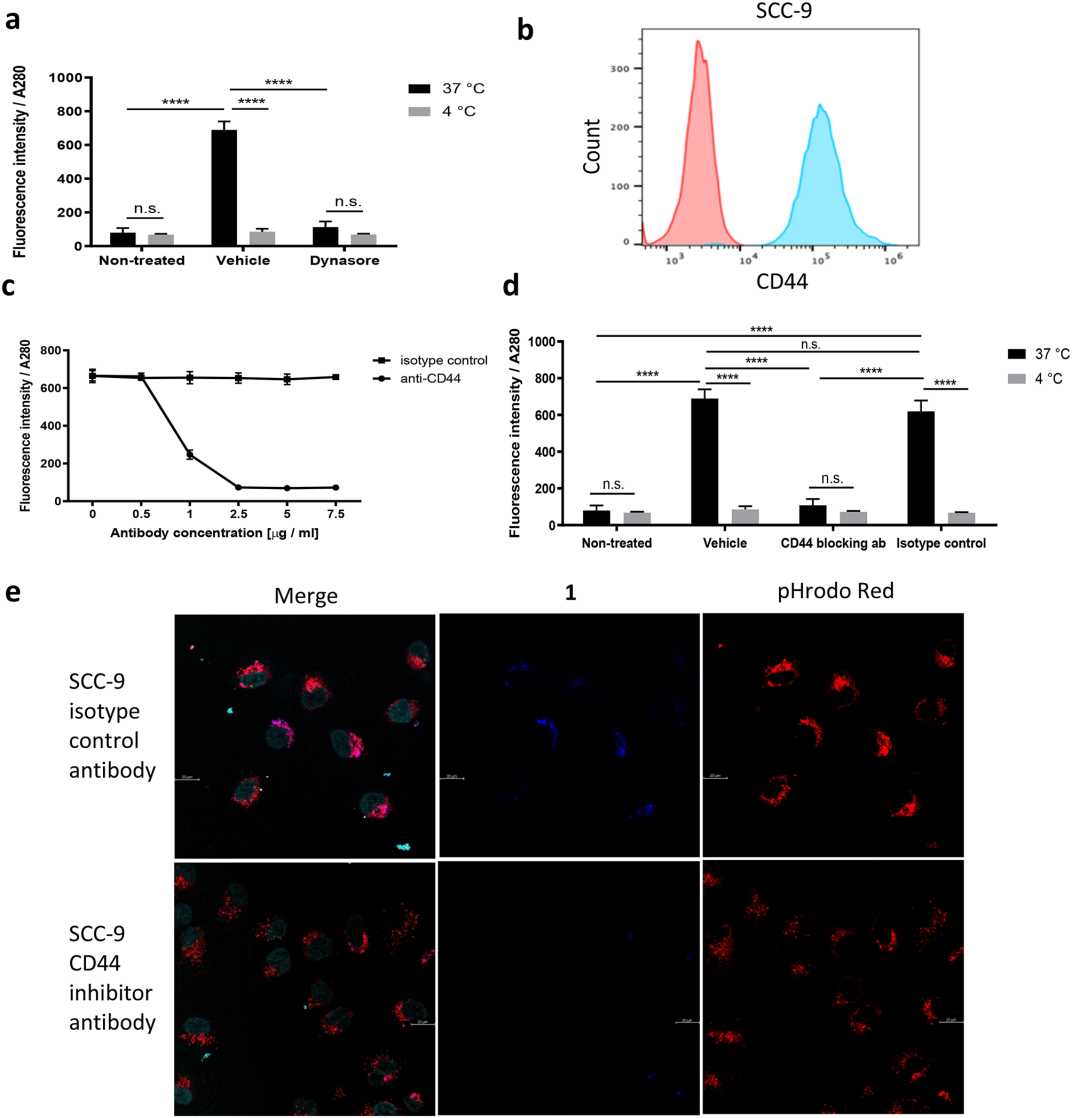
Analysis of the cellular uptake mechanism of compound **1** by SCC-9 cells. (**a**) Inhibition of dynamin related endocytosis and its effect on **1** uptake. Cells were treated with 100 μM Dynasore or 0.1% DMSO as vehicle for 30 min and then co-treated with 5 μM **1** for 1 h at 37 °C and 4 °C. Fluorescence values were corrected for the absorbance at 280 nm. Means ± SD, n = 3. Two-way ANOVA, * *p* < 0.05, ** *p* < 0.01, *** *p* < 0.001, **** *p* < 0.0001, n.s. not significant. (**b**) Expression of CD44 receptor by SCC-9 cells as analysed by FlowJo Software^22^. A representative histogram, showing the level of expression of CD44 in SCC-9 cells (blue) compared to isotype control (red). (**c**) Titration of the CD44 antibody for its effect on **1** uptake. Cells were treated with different concentrations of CD44 blocking antibody for 30 min and then co-treated with **1** (5 μM) for 1 h. Fluorescence of the cell lysates was corrected against the absorbance at 280 nm. Means ± SD, n = 3. (**d**) Inhibition of CD44 receptor mediated endocytosis of **1** by SCC-9 cells. Cells were treated with 2.5 μg/mL antiCD44 antibody for 30 min and then co-treated with **1** (5 μM) for 1 h at 37 °C and 4 °C. Fluorescence of the cell lysate was corrected against the absorbance at 280 nm. Means ± SD, n = 3. Two-way ANOVA, **** *p* < 0.0001, n.s. not significant. Graphs were plotted using GraphPad version 6.0c for Mac, www.graphpad.com. (**e**) Inhibition of CD44 receptor mediated endocytosis and colocalization of **1** with the endocytic vesicles. Cells were treated with 2.5 μg/mL CD44 blocking antibody or isotype control antibody for 30 min, then cotreated with 20 μM **1** for 2 h, SYTO Deep Red Nucleic Acid Stain for 1 h, and 30 μg/mL pHrodo Red for 30 min in serum free medium. Cells were then washed twice with PBS and fresh serum free medium added immediately prior to imaging. Bar is 20 μm. Images were analysed using Leica Application Suite X version 3.5.5.19976, www.leica-microsystems.com.

To investigate the energy dependent uptake of **1**, SCC-9 cells were pre-treated with dynasore—an inhibitor of the dynamin GTPase activity that is required for the formation of clathrin coated vesicles during receptor mediated endocytosis—for 30 min prior to the addition of **1**. It was found that **1** fluorescence was significantly decreased in SCC-9 cell lysates after dynasore treatment in agreement with a dynamin related endocytic mechanism (Fig. 3a). Given that **1** contains a low molecular weight HA backbone, the possibility was investigated that the compound may be recognised and internalised by the CD44 receptor, a transmembrane HA receptor that is dependent on clathrin-mediated uptake.

Towards addressing this possibility, flow cytometry was used to confirm high levels of CD44 expression in SCC-9 cells, with approximately 99% of the cell population staining positive as found by our FlowJo Software^22^, relative to cells treated with isotype control antibody (Fig. 3b). Subsequently, an unconjugated IM7 rat monoclonal anti-CD44 antibody was used to inhibit CD44 mediated endocytosis of **1** across a range of concentrations (Fig. 3c). A near complete block of the uptake of **1** (~95%) was observed at antibody concentrations greater than 2.5 µg/mL. This contrasts with the inability of an unconjugated isotype control rat IgG2b kappa antibody to inhibit **1** uptake, confirming that the exclusive means of **1** internalisation is by endocytosis through the CD44 receptor. Furthermore, as expected, exposing cells pre-treated with CD44 blocking antibody to **1** at 4 °C failed to result in uptake of the probe (Fig. 3d).

To provide further confirmation that **1** is exclusively taken up into intracellular vesicles in a CD44 dependent manner, confocal imaging was performed on **1** in SCC-9 cells in conjunction with the live cell endocytosis tracer pHrodo Red Dextran (10,000 MW dextran whose fluorescence intensity increases with an increase of acidity) and the nuclear stain SYTO Deep Red Nucleic Acid Stain (cell-permeant nuclear stain). Notably, **1** was found to co-localise with pHrodo Red, and is exclusively located in vesicles with an absence of staining in the cellular cytoplasm (Fig. 3e). In addition, treatment of SCC-9 cells with an inhibitory antibody to CD44 led to a pronounced decrease in **1** uptake corroborating our earlier data showing the importance of the endocytic system in internalising **1**.

**Figure 4:**
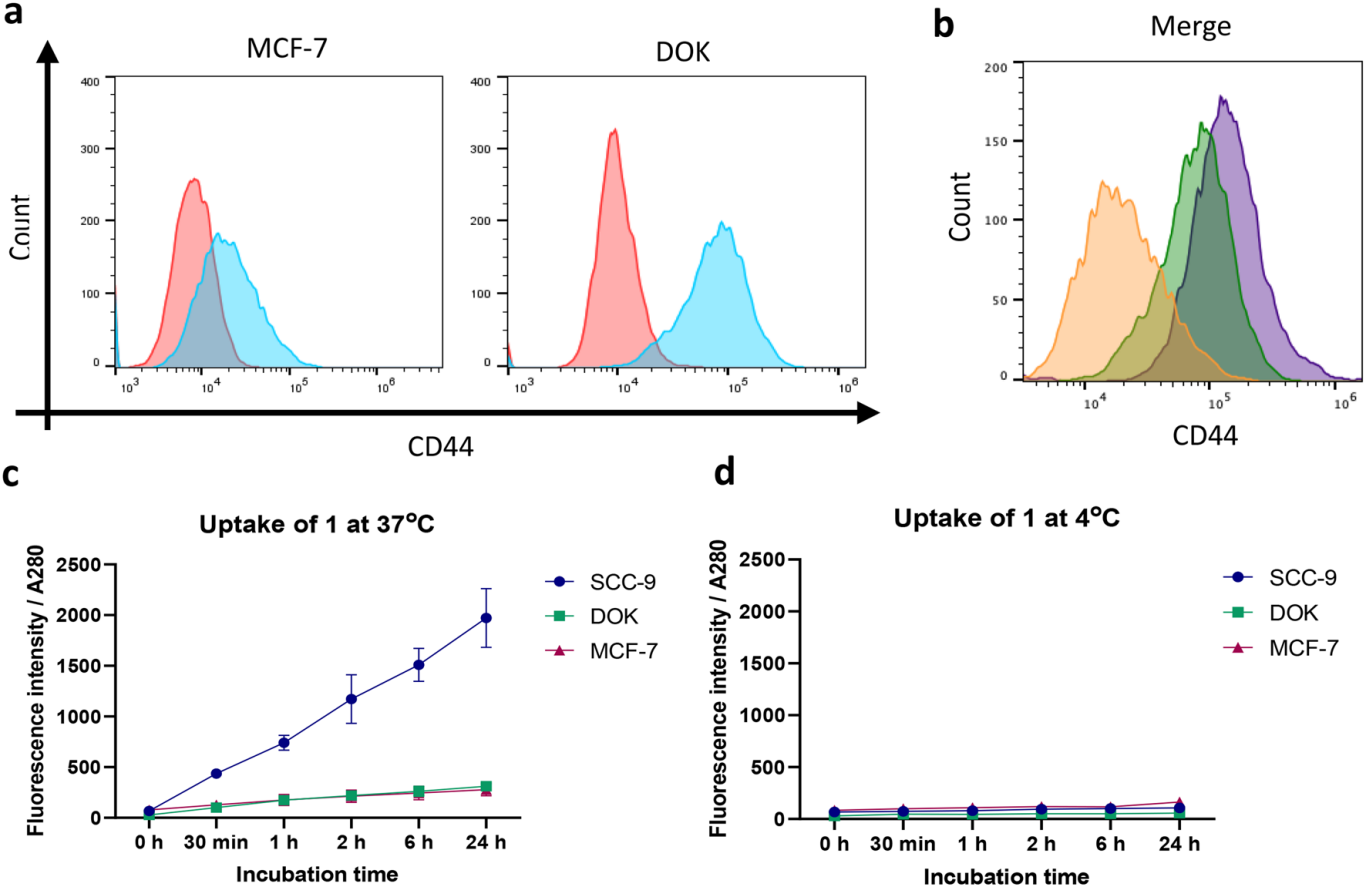
Uptake of **1** in dysplastic and poorly tumorigenic cells. (**a**) A representative histogram, showing the level of expression of CD44 in DOK (dysplastic, non-tumorigenic) and MCF-7 (poorly tumorigenic) cells analysed by FlowJo Software^22^. 1.5 × 10^5^ cells grown in normal culture medium were harvested, washed in PBS, and stained with either anti-CD44 antibody (blue) or isotype control (red) and analysed by flow cytometry. (**b**) A comparative histogram of CD44 expression levels in MCF-7 (orange), DOK (green), and SCC-9 (purple) cells as analysed by FlowJo Software^22^. (**c**,**d**) Analysis of time-dependent uptake of **1** by SCC-9, DOK, and MCF-7 cells. Cells were seeded on a 6-well plate at 3 × 10^5^ cells/well and serum starved for 16 h, and then treated with 5 μM **1** for 24 h, 6 h, 2 h, 1 h, and 30 min at 37 °C, or 4 °C. Cells were then washed twice with PBS and lysed. The fluorescence of the cell lysates was analysed at an excitation of 325 nm and emission of 445 nm. Fluorescence values were corrected for the absorbance at 280 nm. n = 3. Graphs were plotted using GraphPad version 6.0c for Mac, www.graphpad.com.

Ostensibly, as CD44 is overexpressed in a variety of cancer types and is a recognised marker of cancer stem cells, this mode of internalisation should allow facile tumour identification. However, CD44 is also expressed in a number of normal cell types where it participates in cell proliferation, adhesion, and migration^23^. Therefore, we examined the uptake of **1** in the non-tumorigenic, dysplastic DOK cells (oral keratinocytes) and poorly tumorigenic MCF-7 cells (breast cancer) to determine if the label was capable of distinguishing cells based on tumorigenicity. An evaluation of CD44 expression by our FlowJo Software^22^ showed that both cell types expressed the receptor (DOK 69.3 %; MCF-7 7.64 %) (Fig. 4a) albeit at lower levels than SCC-9 cells (99 %), based on mean fluorescence intensity (Fig. 4b). The low CD44 expression seen in MCF-7 cells lead to an expected decrease in **1** uptake but significantly, despite the relatively high presence of CD44 on DOK cells, the uptake of **1** was only approximately one-seventh that of SCC-9 (Fig. 4c,d, Supplementary Fig. S4). These observations highlight the importance of intracellular uptake and align with the recent appreciation that endocytosis is enhanced and skewed in cancer cells and is intimately linked to the tumorigenic and metastatic state^24, 25^.

### Compound 1 lacks cytotoxicity

To evaluate the effect on cell proliferation and viability, Alamar Blue assays were performed with SCC-9 cells exposed to increasing concentrations of **1** and its constituent components for 24 h (Supplementary Fig. S5). The hyaluronan based building blocks of **1** did not show toxicity in SCC-9 cells up to a concentration of 100 μM. Interestingly, OA had a negative impact on the viability of SCC-9 cells at comparatively lower concentrations^26, 27^, with an IC_50_ value of 19.7 μM. By contrast, **1** exhibited only low observable toxicity in SCC-9 cells at a concentration of 100 μM, with cell viability remaining above 80% relative to vehicle control. Notably, this concentration is 20-fold higher than that required for fluorescence detection studies (5 µM) and represents a broad therapeutic window for the use of this agent clinically (Supplementary Fig. S5).

**Figure 5:**
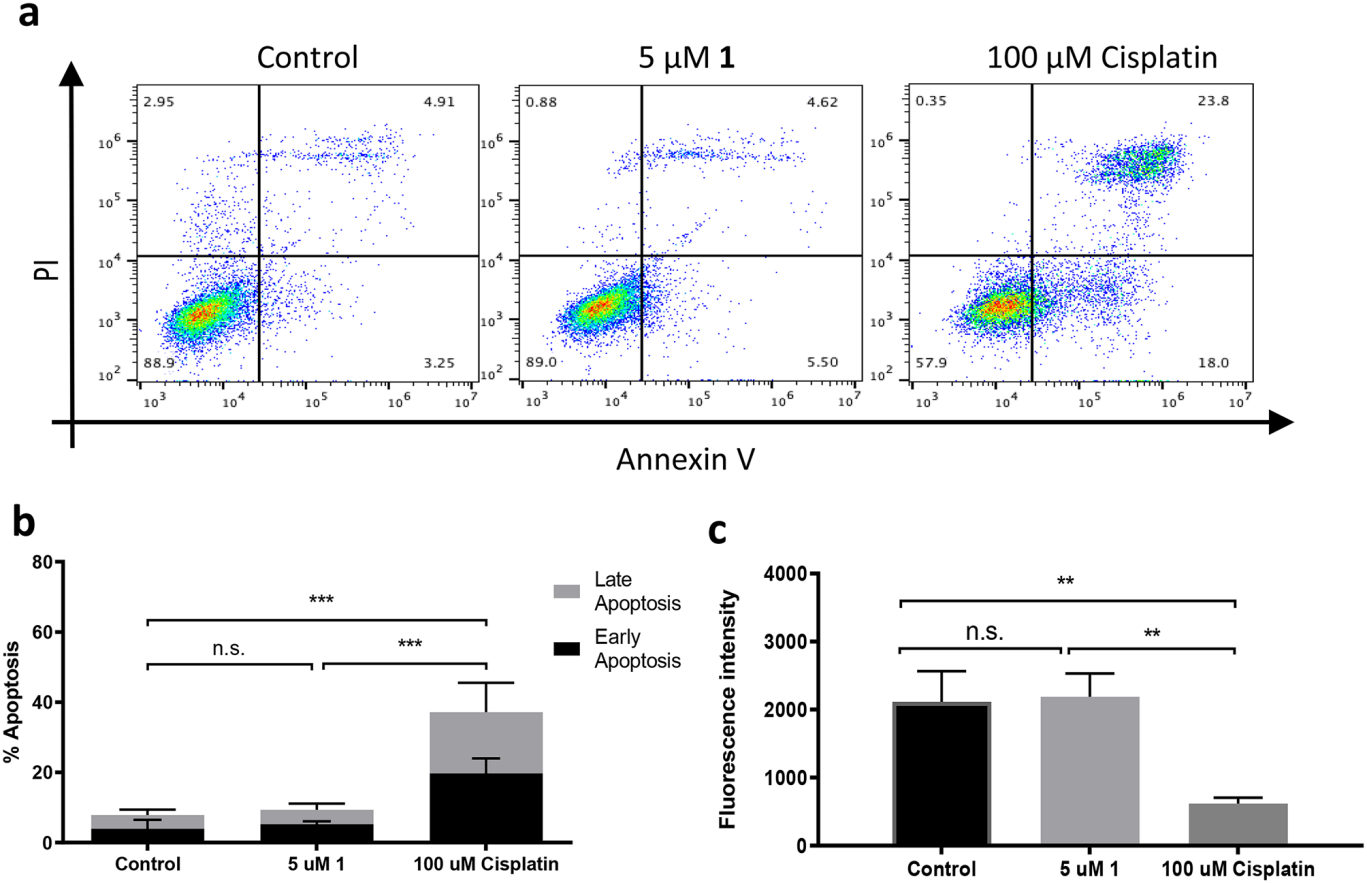
Analysis of compound **1** toxicity in SCC-9 cells. (**a**) A representative dotplot of flow cytometric analysis of cell toxicity of **1** analysed by FlowJo Software^22^. After 24 h of treatment with compound **1** or positive control (Cisplatin), cells were harvested, stained with Annexin V-FITC and propidium iodide, and analysed by flow cytometry. (**b**) Quantitation of apoptotic levels after treatment; means ± SD, n = 3. Two-way ANOVA, *** *p* < 0.001, n.s. not significant. (**c**) Viability of SCC-9 cells after 24 h treatment with **1,** assessed by Alamar Blue assay. Means ± SD, n = 3. Student *t* test, ** *p* < 0.01, n.s. not significant. Graphs were plotted using GraphPad version 6.0c for Mac, www.graphpad.com.

Next, the effect of the working concentration of **1** (5 µM) on cellular apoptosis in SCC-9 cells was investigated relative to the effect of cisplatin and vehicle treatment (Fig. 5a–c). Since the SCC-9 cells have been reported to show resistance to cisplatin^28^, a concentration of 100 μM was chosen to observe an apoptotic effect. As expected, SCC-9 cells treated with cisplatin presented significantly increased (~37%) levels of Annexin V and PI positive staining as found by our FlowJo Software^22^ (*p*<0.001, Two-way ANOVA) (Fig. 5a,b). By contrast, **1** treated SSC-9 cells displayed basal levels of Annexin V and PI staining, comparable to the level of vehicle treated controls, suggesting no induction of apoptosis. The observed lack of cell death induction confirms the non-toxic nature of **1** and underlines its potential utility as a cancer cell imaging agent.

## Discussion

Effective treatment of oral cancer remains largely reliant on surgical intervention. Evidently, the location of tumours within the oral-facial region calls for a high degree of precision in selecting cancerous tissue over normal tissue to minimise disfigurement or co-morbidities at such a critical site in the body. Current techniques to facilitate accurate tissue removal during surgery include non-targeted fluorescent dyes such as ICG, 5-ALA or tissue autofluorescence, and other recent methodologies that are in development such as fluorescently labelled PARP1 and EGFR ligands^13, 14, 29–32^. Here, we describe the synthesis and characterisation of a water soluble, non-toxic cancer imaging probe, referred to as **1**, that is derived exclusively of natural components, and which holds promise as an intraoperative imaging agent in oral cancer.

The backbone of **1** was designed around a HA molecule, an essential mucopolysaccharide that plays a key role in cancer progression and metastasis^33–37^. *In vivo*, HA is recognised by the CD44 receptor, a recognised cancer stem cell marker that is highly expressed on the plasma membrane of oral cancer cells and a range of other cancer types^38–48^. Our data shows that **1** is internalised by clathrin-mediated endocytosis through specific interaction with the CD44 receptor despite the significant FA-OE sidechain modifications^49^. Importantly, **1** does not interact with the cell membrane, as no observable cell associations was found to occur when cells were incubated at 4 °C.

Markedly lower levels of **1** accumulation were observed in cells with low tumorigenicity (MCF-7 breast cancer cells) and low CD44 expression or which are dysplastic and are unable to form tumours (DOK oral keratinocytes) despite the latter expressing reasonable levels of CD44 receptor. This suggests that, in addition to CD44 expression, the uptake of **1** is related to the tumorigenic status of the cancer cells. Indeed, chemically-derivatised conjugates of hyaluronic acid have been prepared with a range of chemotherapeutic agents and have shown selectivity towards tumour cell uptake and were effective in animal models of cancer^50^. For example, Taxol-HA was found to be cytotoxic to a range of tumour cell lines of breast, colon, and ovarian origin but not human fibroblasts and paclitaxel-HA and butyrate-HA conjugates were found to inhibit tumour growth and reduce metastasis in mice^51–53^. A likely explanation for the specificity shown by HA-derivatives, including **1,** is an enhanced level of endocytosis and receptor recycling which is a functional feature of cancer cells and that is known to have a significant role in tumour progression and metastasis^24, 25, 54, 55^.

Confocal and multiphoton imaging confirmed that **1** is present in discrete intracellular vesicles and together with medium dilution studies, showed that the compound is not dispersed in the cytoplasm of the cell, even after prolonged treatment times of 24 h. The vesicular accumulation has important consequences for the use of **1** as a cancer imaging agent, due to fluorescence inducible upon aggregation, commonly referred to AIE. Fluorogens that demonstrate AIE are weakly emissive in solution, however, their emission is greatly increased upon aggregation resulting from restriction to their intramolecular motion^56^. An important feature of AIE for tumour imaging is the ability to use high concentrations and yet avoid background fluorescence, self-quenching, or photobleaching^56–59^. We speculate that the presence of oleic acid in the polymer molecule grants **1** appropriate amphiphilic properties to self-assemble and form aggregates in an aqueous environment and the observed entrapment in endocytic vesicles could further aid the aggregation process, providing yet higher imaging resolution^17^.

The FA moiety, which is the fluorescently active group of **1**, is universally present in plant cell walls, where it is the main fluorophore exhibiting blue-green fluorescence upon excitation with ultraviolet light^19, 60^. It has an interesting Stokes shift of circa 100 nm, which can be further modified by the pH and polarity of the solvent^19, 61, 62^. We have observed a high intensity of fluorescence of **1** by multiphoton microscopy, a method that utilises two or more low energy photons to excite the fluorophore simultaneously, and through a summation of their energy, results in fluorescence^20^. An excitation wavelength of 730 nm would ensure deep penetration into the dermal layers of oral tissue and the use of low energy, long wavelength deep red visible light allows for safe tissue imaging, without the induction of photodamage, that would typically require the use of shorter wavelengths for excitation^63^. Currently, the clinical use of multiphoton microscopy is mainly limited to assessing tissue biopsies and multiphoton tomography^64, 65^. However, multiple *in vivo* skin imaging multiphoton systems are being developed for commercial use, that conceivably could be used for microendoscopy in the oral cavity^65–70^.

Bio-adhesive patches and hydrogels have been used for many decades for the *in-situ* delivery of compounds to the periodontal regions and oral mucosa, including in oral cancer^71–76^. Adoption of such systems could enable topical application of **1** on suspected or confirmed tumours, providing sufficient time for the probe to accumulate prior to surgery. The aqueous solubility and lack of observable toxicity, together with the fact that the uptake of **1** is fully reversible would ensure that the imaging agent could be expelled from the body without systemic accumulation. Further studies are ongoing to evaluate the topical application of **1** for oral cancer imaging.

## Methods

### Spectroscopy

The absorption spectrum of **1** was obtained using a SPECTRAmax PLUS 384 Microplate Spectrophotometer (Molecular Devices). Emission spectrum was obtained with a SPECTRAmax Gemini Microplate Spectrofluorometer (Molecular Devices), correcting for the solvent response.

### Cell culture

Frozen stocks of SCC-9, DOK, and MCF-7 cells were purchased from Sigma Aldrich. Cells were cultured at 37 °C, in a humidified atmosphere, containing 5% CO_2_. For routine culture of SCC-9 cells, DMEM/F-12 medium (Sigma Aldrich) supplemented with 10% Foetal Bovine Serum (FBS), 2.5 mM L-Glutamine, 0.5 mM Sodium Pyruvate, 400 ng/mL hydrocortisone, and 1% Penicillin/Streptomycin (Gibco) was used. DOK cells were cultured in DMEM medium supplemented with 10% FBS, 2 mM L-Glutamine, 5 μg/mL hydrocortisone, and 1% Penicillin/Streptomycin. MCF-7 cell culture was maintained in DMEM medium supplemented with 10 % FBS, 2 mM L-Glutamine, 1% Non-Essential Amino Acids (NEAA), 1% Insulin-Transferrin-Selenium, and 1% Penicillin/Streptomycin (Gibco).

### Cytotoxicity assays

SCC-9 cells were plated on clear, flat-bottomed 96-well plates at 5 × 10^3^ cells/well and left in routine medium to attach (~6-8 h). The medium was changed to FBS free conditions to serum starve the cells for 16 h before performing the assay. After 16 h of serum starvation, cells were treated with a range of concentrations of the tested compounds (0.24 μM to 100 μM final), in a volume of 100 μL serum free medium. Vehicle control was MilliQ H_2_O diluted in medium. Each concentration of compound was added in triplicate. After 24 h at 37 °C and a humidified atmosphere of 5% CO_2_, cell viability was assessed using Alamar Blue (Invitrogen) viability assay in comparison to vehicle control. IC_50_ values were determined using GraphPad version 6.0c for Mac, GraphPad Software, San Diego, California USA, www.graphpad.com, from 3 independent biological replicates for each experiment.

### Flow cytometry assays

Annexin V/Propidium Iodide double staining was used to analyse apoptotic levels in SCC-9 cells after 1 treatment. Early apoptotic cells stain positive for Annexin V and negative for PI, while late apoptotic cells stain positive for both, Annexin V and PI. In this study, Annexin V-FITC and PI were used. Cells were seeded on 6-well plates, at a density of 1.5 × 10^5^ cells/well, serum starved for 16 h and treated for 24 h with either 5 μM 1, 100 μM cisplatin, or an appropriate volume of MilliQ H_2_O as vehicle control. Cells were harvested, pelleted, washed with PBS, and stained with Annexin V-FITC (IQ Products) (1:25 dilution in Annexin V binding buffer) on ice for 20 min. Then, cells were centrifuged for 5 min at 250 × g, supernatant removed, and 1:400 PI (Invitrogen) dilution in Annexin V binding buffer was added. Cells were analysed by flow cytometry using BD Accuri C6 (BD Biosciences) software, reading 10,000 events per sample, with further analysis being performed with FlowJo version 10.6 software^22^. The percentages of early apoptotic and late apoptotic cell populations from three independent experiments were plotted in GraphPad Prism version 6.0c for Mac, GraphPad Software, San Diego, California USA, www.graphpad.com.

FITC conjugated IM7 rat monoclonal anti-CD44 antibody and a FITC conjugated rat IgG2b kappa monoclonal isotype control antibody (Invitrogen) were used to quantify CD44 receptor expression in SCC-9, DOK, and MCF-7 cells. Cells were grown in standard culture medium and harvested and counted as described above. 1.5 × 10^5^ cells were washed in PBS and stained with either anti-CD44 antibody or isotype control antibody (1:400 dilution in 1% FBS in PBS) on ice for 20 min. Cells were centrifuged for 5 min at 250 × g and resuspended in 100 μL of 1:400 PI diluted in 1% FBS in PBS. Cells were then analysed by flow cytometry using BD Accuri C6 software reading 10,000 events per sample, with further analysis using FlowJo version 10.6 software^22^. Cell populations from three independent experiments were gated, and CD44 positive cells plotted against isotype control.

### Cellular uptake studies

SCC-9, DOK, and MCF-7 cells were seeded and serum starved as described above. Further dilutions of compound **1** stock solution were performed in serum-free medium. Cells were treated with 5 μM of compound **1** for 24 h, 6 h, 2 h, 1 h, and 30 min either at 37 °C or 4 °C to distinguish non-specific binding to the plasma membrane from active uptake. A vehicle control of MilliQ H_2_O was included for each assay. The cells were collected and processed as described above, and the fluorescence levels measured using a SPECTRAmax M3 Microplate Spectrofluorometer with an excitation of 325 nm and emission of 445 nm. Fluorescence values were corrected for A_280_ values measured using a SPECTRAmax M3 Microplate Spectrofluorometer. The absorbance and fluorescence values for the solvent were subtracted from the samples as baseline.

### Endocytic inhibition studies

Dynasore, an inhibitor of dynamin GTPase activity, were used in endocytic assays. SCC-9 cells were seeded and serum starved, as described above, and either left untreated, or treated with 100 μM dynasore or vehicle (0.1% DMSO) for 30 min. Then, either 5 μM **1** or a vehicle control of MilliQ H_2_O were added to the cells for 1 h in conjunction with the inhibitors. Cells were incubated at either 37 °C or 4 °C to distinguish energy dependent uptake from non-specific binding to the plasma membrane. Cells were harvested, lysed using a 25-gauge needle, centrifuged and the fluorescence and A_280_ values measured in supernatants by SPECTRAmax Gemini Microplate Spectrofluorometer and SPECTRAmax PLUS 384 Microplate Spectrophotometer, respectively. Absorbance and fluorescence values for the solvent were subtracted from the samples as baseline. Three independent experiments were performed for each condition.

### CD44 uptake studies

The effect of blocking CD44 on the uptake of **1** in SCC-9 cells was investigated using an unconjugated IM7 rat monoclonal anti-CD44 antibody and an unconjugated rat IgG2b kappa monoclonal isotype control antibody (Invitrogen). First, a saturation curve for the blocking antibody was performed. Cells were seeded as described above, and then treated with either an appropriate volume of MilliQ H_2_O as vehicle or a range of concentrations (0.5 μg/mL–7.5 μg/mL) of the anti-CD44 and isotype control antibodies for 30 min and co-treated with 5 μM of compound **1** for a further 1 h at 37 °C. The cells were then collected and processed, and the fluorescence and A_280_ levels were measured as described above. The absorbance and fluorescence values for the solvent were subtracted from the samples as baseline. Three independent experiments were performed for each condition. Inhibition of uptake was observed at 2.5 μg/mL. Finally, cells were seeded as described above, and then were either left untreated or treated with either an appropriate volume of MilliQ H_2_O as vehicle or 2.5 μg/mL of the anti-CD44 and isotype control antibodies for 30 min and subsequently co-treated with 5 μM of compound **1** for a further 1 h at 37 °C or 4 °C to distinguish uptake from non-specific binding to the plasma membrane. The cells were then collected and processed, and the fluorescence and A_280_ levels were measured as described above. The absorbance and fluorescence values for the solvent were subtracted from the samples as a blank. Three independent experiments were performed for each condition.

### Confocal microscopy

For the initial study on SCC-9 cells, c cells were seeded onto an 8 chamber μ-slide with cover slip base (ibidi) at a density of 1 × 10^5^ cells/chamber. Cells were left to attach and then serum starved for 16 h before treatment. After serum starvation, cells were treated with 20 μM of **1** or vehicle control in serum-free medium and incubated for 2 h. Cells were then washed twice with PBS, and medium was then changed to fresh serum-free standard culture medium, to remove excess compound. To visualize the outlines of the cells and show internalization of **1**, 0.1 μM fluorescein sodium salt was added to the medium immediately prior to visualizing. Cells were then examined on a Leica SP8 Confocal Microscope using Leica Application Suite X (LAS X) version 3.5.5.19976, Leica Microsystems, Germany, www.leica-microsystems.com. 3D rendering was performed using Imaris version 9.5, Oxford Instruments, www.imaris.oxinst.com.

For the confocal study with inhibitor antibody, SCC-9, DOK, and MCF-7 cells were seeded onto an 8 chamber ibidi μ-slide with cover slip base at a density of 1 × 10^5^ cells/chamber. Cells were left to attach and then serum starved for 16 h before treatment. After serum starvation, cells were treated with 2.5 μg/mL of either the anti-CD44 inhibitor antibody or isotype control antibody for 30 min and subsequently co-treated with 20 μM of compound **1** for a further 2 h, 1X SYTO Deep Red Nucleic Acid Stain for 1 h, and 30 μg/mL pHrodo Red for 30 min in serum free medium at 37 °C. Cells were then washed twice with PBS to remove excess staining compounds, and fresh serum-free medium was added. Cells were then examined on a Leica SP8 Confocal Microscope using LAS X version 3.5.5.19976, Leica Microsystems, Germany, www.leica-microsystems.com.

### Multiphoton microscopy

SCC-9 cells were seeded on a 6-channel VI 0.4 μ-slide (ibidi) at a density of 5 × 10^4^ cells/channel. After attachment, cells were serum starved for 16 h prior to treatment. Cells were then treated with 20 μM of **1** in serum-free medium for 2 h. Cells were then washed twice with PBS and fresh serum-free medium was then added to remove excess compound. Cells were then visualized using a custom upright (Olympus BX61WI) laser multiphoton microscopy system equipped with titanium:sapphire laser (Chameleon Ultra, Coherent, USA) and water-immersion 25 × objective (Olympus, 1.05NA) with an excitation of 730 nm and emission between 397 nm and 550 nm. The fluorescence of internalized compound **1** was compared to untreated control. Images were analysed using Olympus Fluoview, FV10 ASW version 4.0, Olympus Corporation, www.olympus-lifescience.com.

### Statistical analysis

Statistical analyses were performed using GraphPad Prism 6.0c version for Mac, GraphPad Software, San Diego, California USA, www.graphpad.com. Results show means ± standard deviation (SD) of three independent experiments. For comparisons of two values, Student t-test was used. For comparison of two or more groups, One-way ANOVA followed by Bonferroni multiple comparison test was performed. For comparison of two or more groups with two independent variables, a Two-way ANOVA test followed by Bonferroni multiple comparison test was performed. Significant values were marked as: * *p* < 0.05, ** *p* < 0.01, *** *p* < 0.001, **** *p* < 0.0001, and n.s. was used to mark not significant values.

## Supplementary Information

Supplementary Information 1.

Supplementary Video 1.
