# Supplementary material for: A non-toxic, reversibly released imaging probe for oral cancer that is derived from natural compounds
Source: Sci Rep. 2021 Jul 7;11:14069. doi: 10.1038/s41598-021-93408-0 (PMC8263592; doi:10.1038/s41598-021-93408-0)
Supplement: Supplementary file 1 — Supplementary Information 1. [file 41598_2021_93408_MOESM1_ESM.docx]

A non-toxic, reversibly released imaging probe for oral cancer that is derived from natural compounds

Magda Ghanim^1^, Nicola Relitti^2^, Gavin McManus^1^, Stefania Butini^2^,*, Andrea Cappelli^2^, Giuseppe Campiani^2^, K. H. Mok^1^ and Vincent P. Kelly^1^,*

^1^ School of Biochemistry & Immunology, Trinity Biomedical Sciences Institute, Trinity College Dublin, Dublin 2, Ireland

^2^ Department of Biotechnology, Chemistry and Pharmacy, Department of Excellence 2018-2022, University of Siena, 53100 Siena, Italy

*Correspondence to butini3@unisi.it and kellyvp@tcd.ie

**SUPPLEMENTARY INFORMATION**

**Chemical synthesis**

All starting chemicals and solvents were purchased from commercial vendors and used without additional purification. Silica gel 60 F254 (0.040–0.063 mm) with UV detection was used for checking the reaction progress. Column chromatography was performed on silica gel 60 (0.040–0.063 mm). ^1^H NMR and ^13^C NMR spectra were recorded on a Varian 300 MHz or Bruker 400 MHz spectrometer. The residual signal of the deuterated solvent was used as internal standard. Splitting patterns are expressed as singlet (s), doublet (d), triplet (t), quartet (q), and broad (br); the value of chemical shifts (d) are given in ppm and coupling constants (J) in hertz (Hz). Mass spectra were recorded utilizing electron spray ionization (ESI). Yields refer to purified products and are not optimized. All reactions were run in an inert atmosphere using oven-dried glassware and anhydrous solvents.

*2,2,3,3-Tetramethyl-4,7,10,13,16,19-hexaoxa-3-silahenicosan-21-ol (****3****).* To a solution of hexa(ethylene glycol) (**2**, 1,00 g, 3.54 mmol) in DCM (30 mL) and DMF (3 mL) cooled at 0 ^o^C, TBSCl (0.480 g, 3.19 mmol), imidazole (0.723 g, 10.62 mmol) and DMAP (6.0 mg, 0.18 mmol) were added. The reaction was allowed to reach 25 ^o^C and stirred for 18 h. After this time, brine (15 mL) was added and the mixture was extracted with DCM (3 x 20 mL). The combined organic extracts were dried over Na_2_SO_4_, filtered, and evaporated. The crude product was purified by flash chromatography on silica gel (2% MeOH in EtOAc) to give the pure tile compound (0.600 g, 43%) as a colourless oil. ^1^H NMR (300 MHz, CDCl_3_) δ 3.78 – 3.47 (m, 24H), 0.86 (s, 9H), 0.03 (s, 6H); MS (ESI) m/z 419 [M + Na]^+^.

*21-Azido-2,2,3,3-tetramethyl-4,7,10,13,16,19-hexaoxa-3-silahenicosane (****4****)*. To a solution of **3** (0.600 g, 1.51 mmol) in dry DCM (10 mL) cooled at 0 ^o^C, TEA (421 µL, 3.02 mmol) and MsCl (234 µL, 3.02 mmol) were added. The reaction was allowed to reach 25 ^o^C and stirred for 2 h. Thereafter, the reaction was washed with brine (10 mL) and the organic layer was dried over Na_2_SO_4_, filtered, and evaporated. The crude material was used in the next step without any further purification. This mesylated product was dissolved in MeCN (10 mL) and DMF (0.5 mL) and NaN_3_ (0.374 g, 4.23 mmol) was added. The reaction was stirred at 85 ^o^C for 18 h then water (10 mL) was added and the mixture was extracted with EtOAc (3 x 10 mL). The combined organic extracts were dried over Na_2_SO_4_, filtered, and evaporated. The crude product was purified by flash chromatography on silica gel (50% PetEt in EtOAc) to give the pure title compound **4** (0.310 g, 49% over two steps) as a colourless oil. ^1^H NMR (300 MHz, CDCl_3_) δ 3.75 (t, *J* = 5.4 Hz, 2H), 3.70 – 3.59 (m, 18H), 3.59 – 3.48 (m, 2H), 3.37 (t, *J* = 5.1 Hz, 2H), 0.88 (s, 9H), 0.05 (s, 6H). MS (ESI) m/z 444 [M + Na]^+^.

*17-Azido-3,6,9,12,15-pentaoxaheptadecyl oleate (****5****)*. To a solution of **4** (0.310 g, 0.74 mmol) in THF (10 mL) cooled at 0 ^o^C, a 1 M solution of TBAF (1.48 mL, 1.48 mmol) in THF was added. The reaction was allowed to reach 25 ^o^C and stirred for 2 h, then brine was added and the mixture was extracted with EtOAc (3 x 10 mL). The combined organic extracts were dried over Na_2_SO_4_, filtered, and evaporated. The crude product was purified by flash chromatography on silica gel (5% MeOH in EtOAc) to give **5** (230 mg, quantitative yield) as a colourless oil. ^1^H NMR (300 MHz, CDCl_3_) δ 3.65 (d, *J* = 1.2 Hz, 22H), 3.42 – 3.31 (m, 2H). MS (ESI) m/z 330 [M + Na]^+^.

Oleic acid (56 µL, 0.18 mmol) was dissolved in THF (3 mL) and CDI (29 mg, 0.18 mmol) was added. The reaction was stirred at reflux for 12 h, then the previously described azido compound (50.0 mg, 0.16 mmol) and DBU (24 µL, 0.16 mmol) were added and the reaction was stirred at 25 ^o^C for 12 h. After this time, the solvent was evaporated and the crude was purified by flash chromatography on silica gel (50% PetEt in EtOAc) to give **4** (30.0 mg, 32%) as a white solid. ^1^H NMR (300 MHz, CDCl_3_) δ 5.39 – 5.18 (m, 2H), 4.26 – 4.09 (m, 2H), 3.77 – 3.54 (m, 20H), 3.36 (t, *J* = 5.1 Hz, 2H), 2.30 (t, *J* = 7.6 Hz, 2H), 2.08 – 1.76 (m, 4H), 1.75 – 1.49 (m, 4H), 1.39 – 1.15 (m, 20H), 0.93 – 0.75 (m, 3H).

MS (ESI) m/z 594 [M + Na]^+^.

*HA-FA-HEG-OE (****1****).* Under an inert atmosphere, a 10 mL flask was charged with *tert*-butanol (2.0 mL), water (2.0 mL), and a solution of CuSO_4_ pentahydrate (12.5 mg, 0.050 mmol) in 0.50 mL of water. A 1 M solution of sodium ascorbate in water (0.50 mL) was then added. 0.5 mL of the resulting mixture was used as the catalyst. A mixture of **5** (30 mg, 0.052 mmol) and HA–FA–Pg-3F (**6**) graft copolymer (100 mg) in water (5.0 mL) was treated with the catalyst solution (0.5 mL) and the reaction mixture was stirred at room temperature for 12 h and then treated with QUADRASIL MP (100 mg). After filtration, the solution was concentrated under reduced pressure. The residue was purified by washing with acetone, then dissolved in H_2_O to allow the polymer to swell. The polymer was then retrieved by centrifugation (4,800 rpm, 3 min, 4 times) giving HA–FA–HEG–OE **1**, which was dried under reduced pressure to obtain as a light brown glassy solid (60 mg). ^1^H NMR (300 MHz, D_2_O) (Supplementary Fig. S2); ^13^C NMR (75 MHz, D_2_O) (Supplementary Fig. S3).

*17-(4-((2-Methoxy-4-((E)-3-methoxy-3-oxoprop-1-en-1-yl)phenoxy)methyl)-1H-1,2,3-triazol-1-yl)-3,6,9,12,15-pentaoxaheptadecyl oleate (****7****).* Starting from **5** (30 mg, 0.052) and methyl (*E*)-3-(3-methoxy-4-(prop-2-yn-1-yloxy)phenyl)acrylate (13 mg, 0.052 mmol) the title compound was obtained following the procedure described for compound **1**. The reaction was quenched with a 5% solution of NH_4_OH (1 mL) and the mixture was extracted with DCM (3 x 5 mL). The combined organic extracts were dried over Na_2_SO_4_, filtered, and evaporated. The crude product was purified by flash chromatography on silica gel (EtOAc) to give title compound (10 mg, 24%) as a white solid. ^1^H NMR (300 MHz, Chloroform-*d*) δ 7.84 (s, 1H), 7.60 (d, *J* = 16.0 Hz, 1H), 7.13 – 6.93 (m, 3H), 6.29 (d, *J* = 16.0 Hz, 1H), 5.48 – 5.13 (m, 2H), 4.52 (t, *J* = 5.0 Hz, 2H), 4.28 – 4.07 (m, 2H), 3.98 – 3.82 (m, 5H), 3.78 (s, 3H), 3.73 – 3.45 (m, 18H), 2.30 (t, *J* = 7.6 Hz, 2H), 1.99 (q, *J* = 8.7, 7.6 Hz, 4H), 1.64 (d, *J* = 29.5 Hz, 4H), 1.46 – 1.09 (m, 20H), 0.98 – 0.67 (m, 3H). ^13^C NMR (75 MHz, CDCl_3_) δ 173.8, 167.6, 149.7, 149.6, 144.6, 143.5, 130.0, 129.7, 127.9, 124.4, 122.4, 115.7, 113.5, 110.0, 70.6, 70.5, 70.5, 70.4, 69.4, 69.2, 63.3, 62.8, 55.9, 53.4, 51.6, 50.3, 34.2, 31.9, 31.9, 29.7, 29.7, 29.6, 29.6, 29.5, 29.3, 29.3, 29.2, 29.1, 29.1, 27.2, 27.1, 24.9, 22.7, 14.1. MS (ESI) m/z 840 [M + Na]^+^.

**SUPPLEMENTARY FIGURES**

**a**

**b**

**Supplementary Figure S1.** Synthesis of Compounds **1** and **5 (Panel a)**, and of compound **7 (Panel b).**

Panel drawn using ChemDraw version 4.5, PerkinElmer Informatics, www.informatics.perkinelmer.com


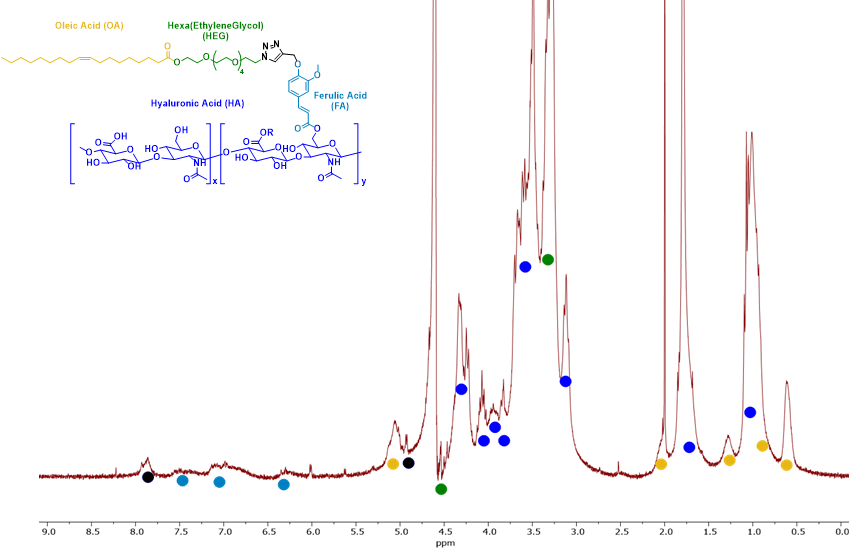


**Supplementary Figure S2.** ^1^H NMR spectrum of compound **1** (in D_2_O)

Yellow dots in the NMR profile indicate the chemical shift of the functional groups in the OA moiety, green dots mark the glycol component, black dots represent 1,2,3-triazole, turquoise dots correspond to the ferulic acid residue, and blue dots point to the hyaluronic acid chains. Chemical structures drawn using ChemDraw version 4.5 software, PerkinElmer Informatics, www.informatics.perkinelmer.com. NMR spectra analysed by MestreNova version 8.1 software, Mestrelab Research S.L. www.mestrelab.com.


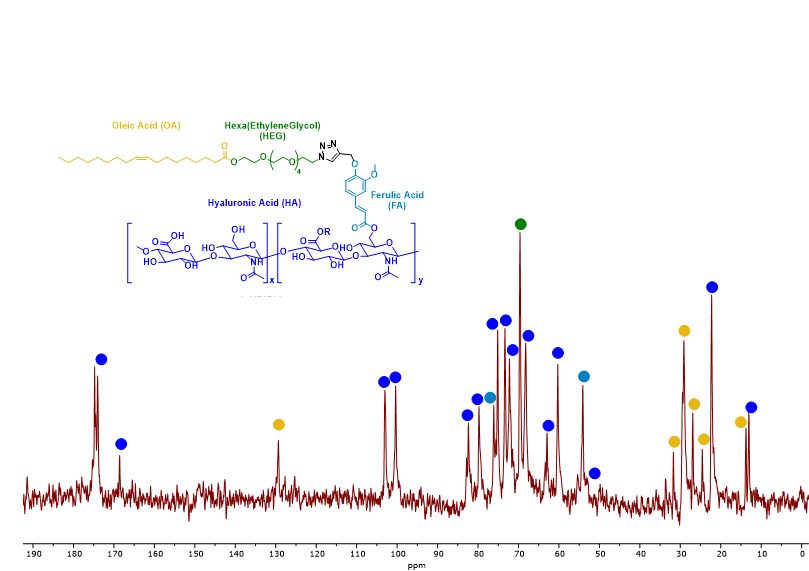


**Supplementary Figure S3.** ^13^C NMR spectrum of compound **1** (in D_2_O)

Yellow dots in the NMR profile indicate the chemical shift of carbon atoms in the OA moiety, green dots mark the glycol component, turquoise dots correspond to the ferulic acid residue, and blue dots point to the hyaluronic acid chains. Chemical structures drawn using ChemDraw version 4.5 software, PerkinElmer Informatics, www.informatics.perkinelmer.com. NMR spectra analysed by MestreNova version 8.1 software, Mestrelab Research S.L. www.mestrelab.com.

Merge

**1**

pHrodo Red

DOK

isotype control

antibody


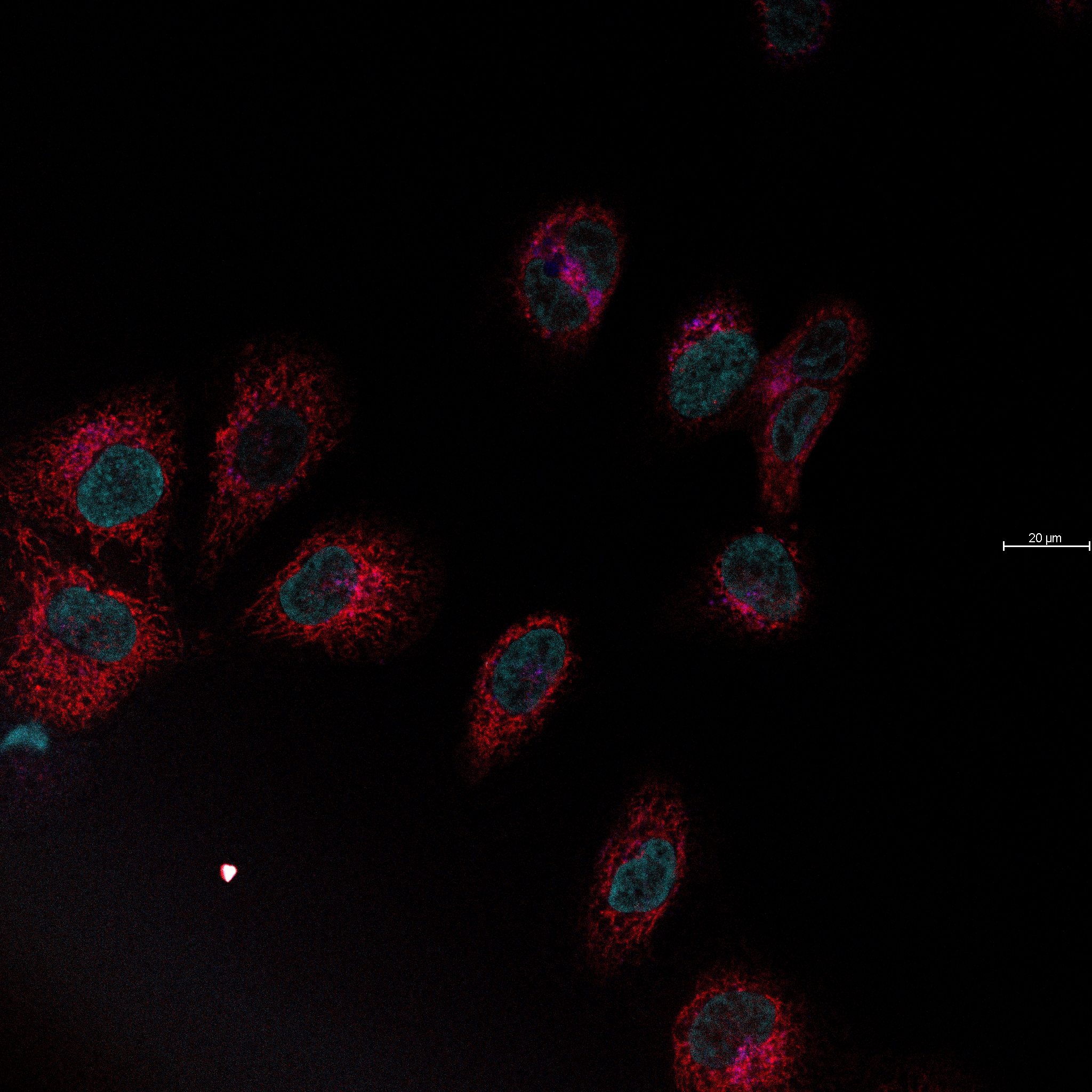

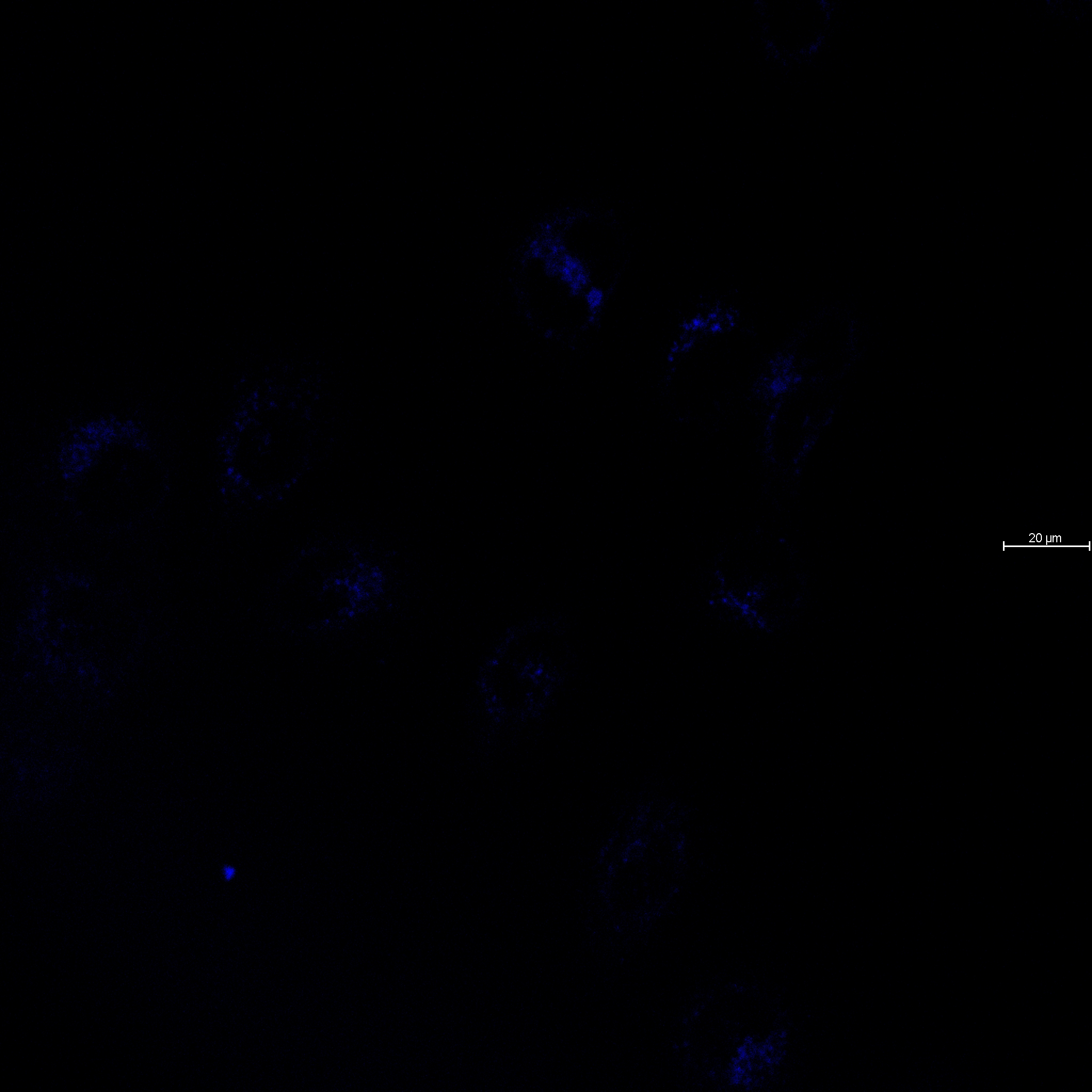

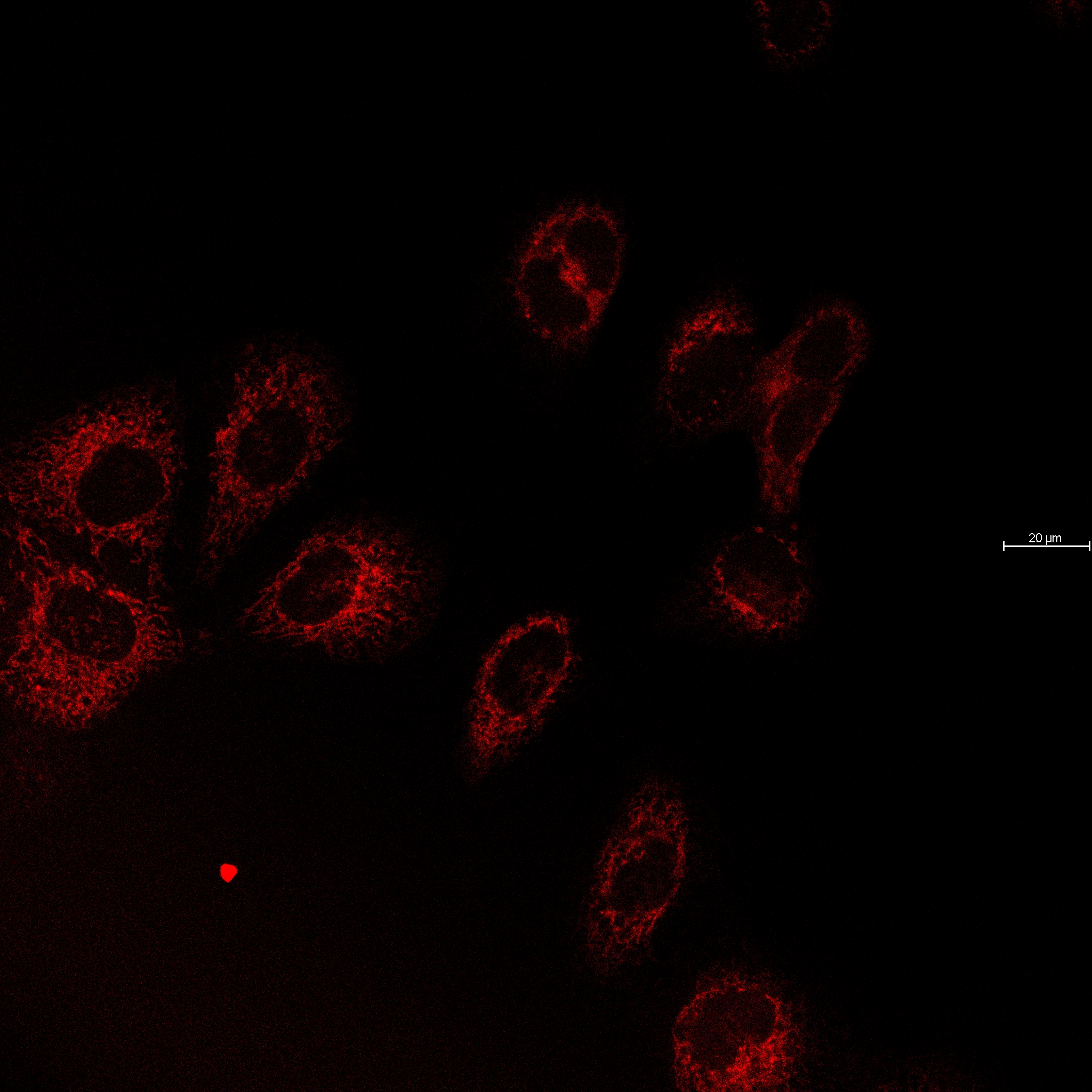


DOK

CD44 inhibitor antibody


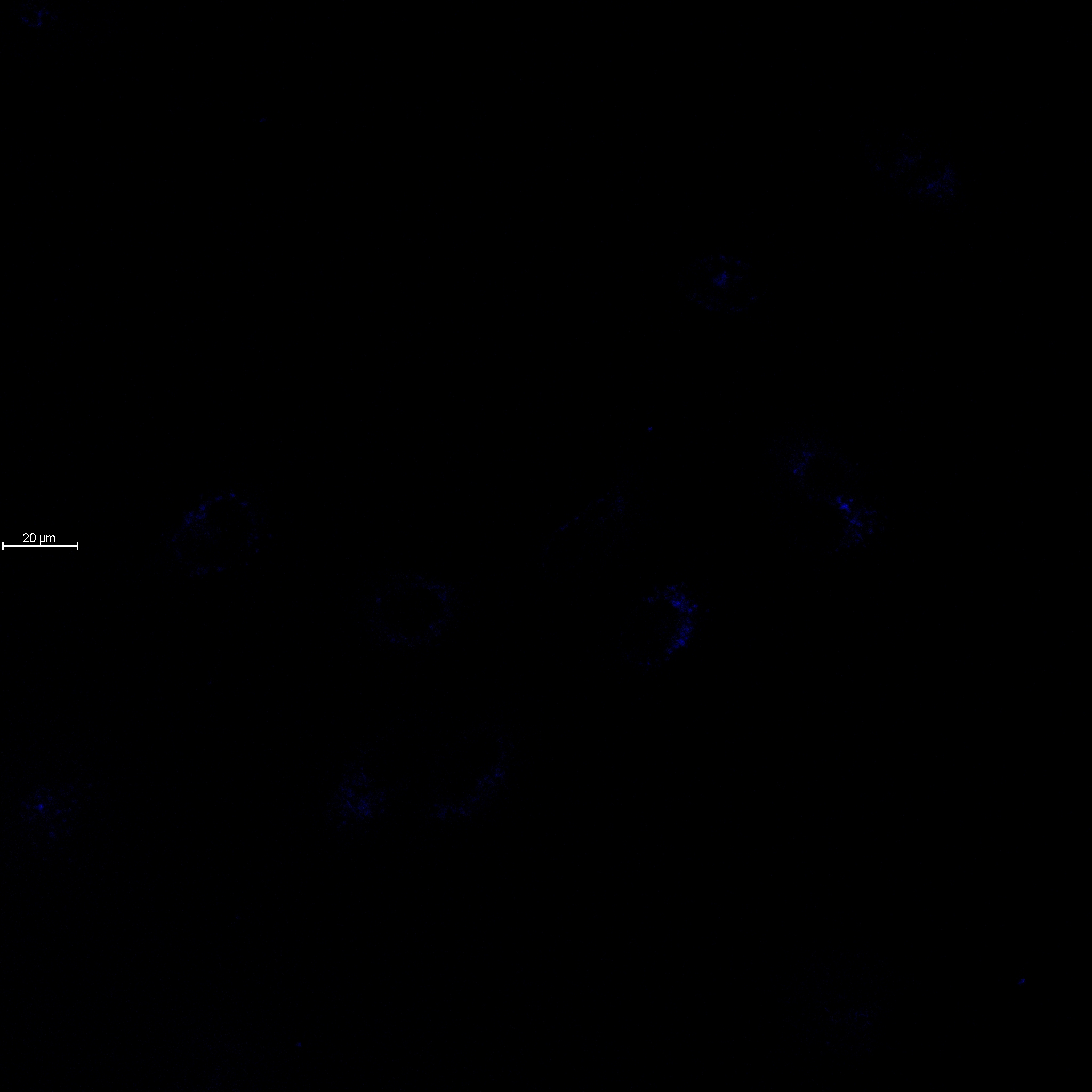

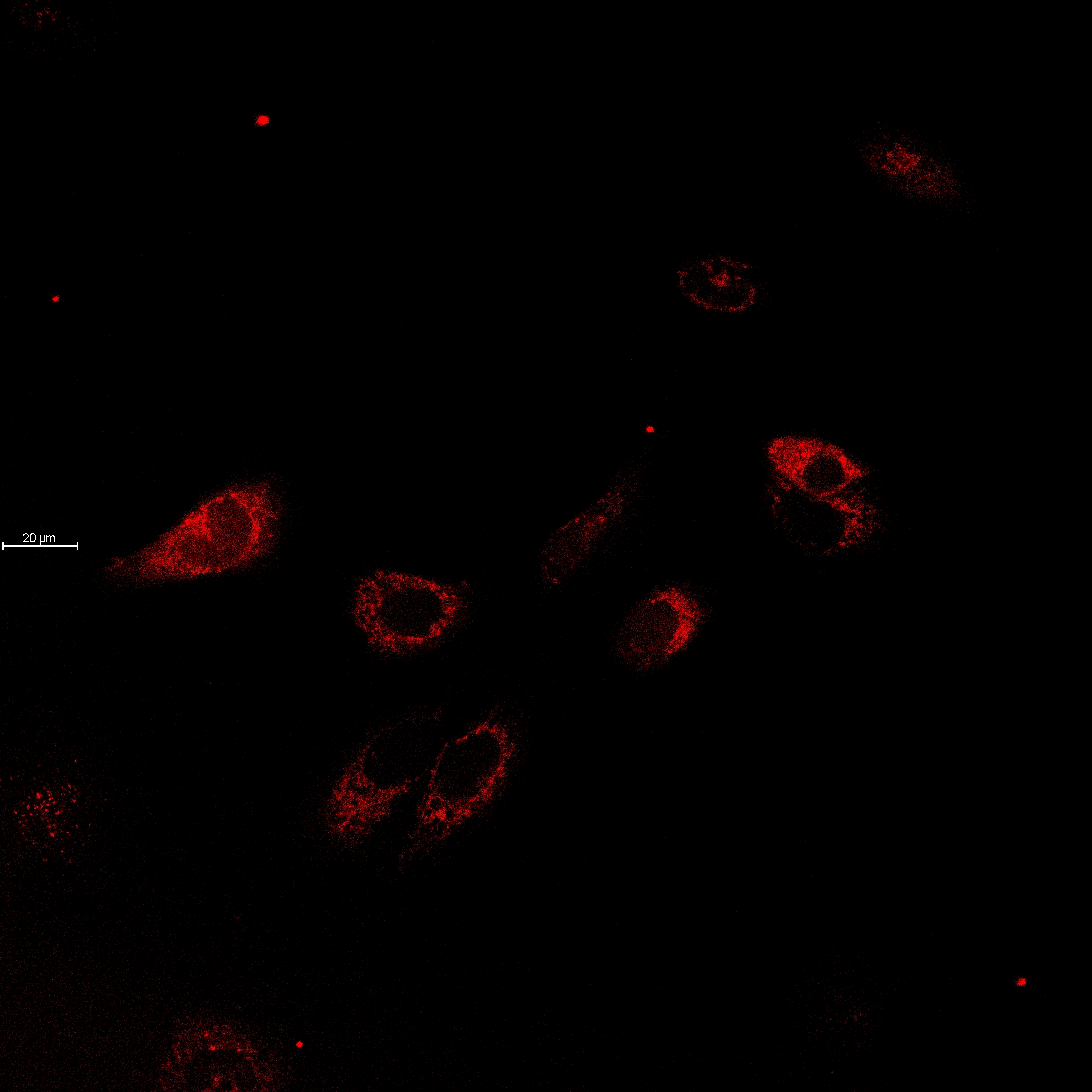

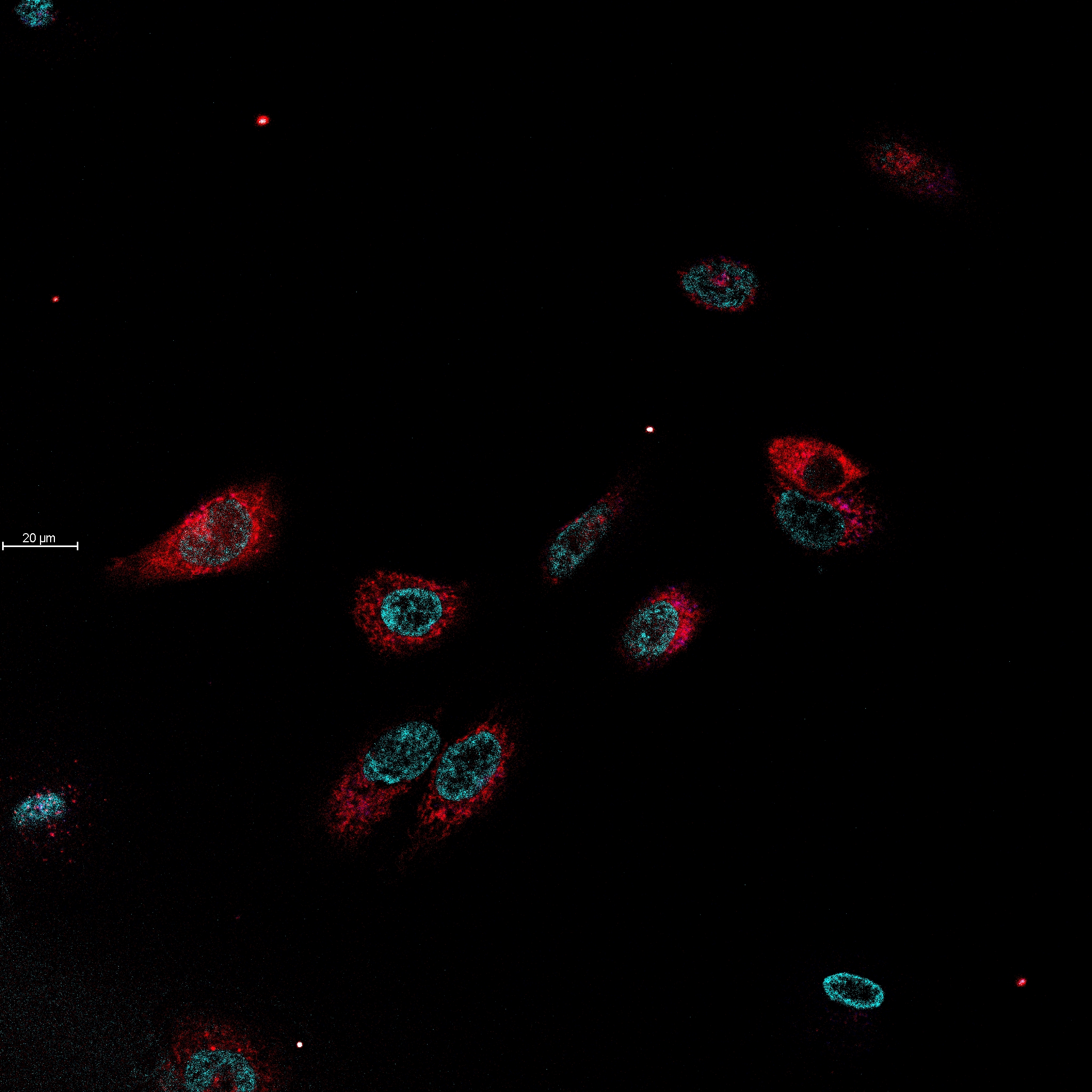


MCF-7

isotype control

antibody


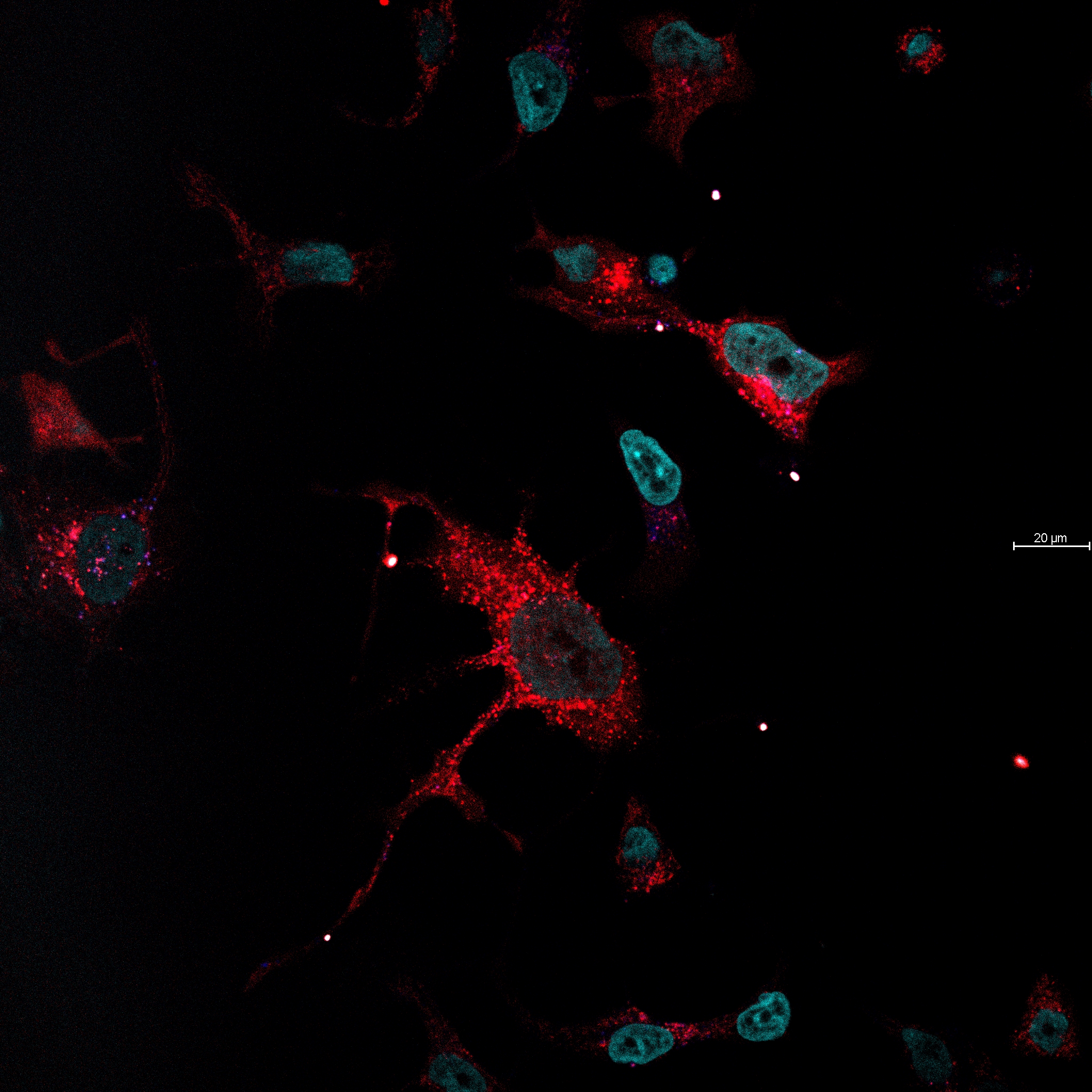

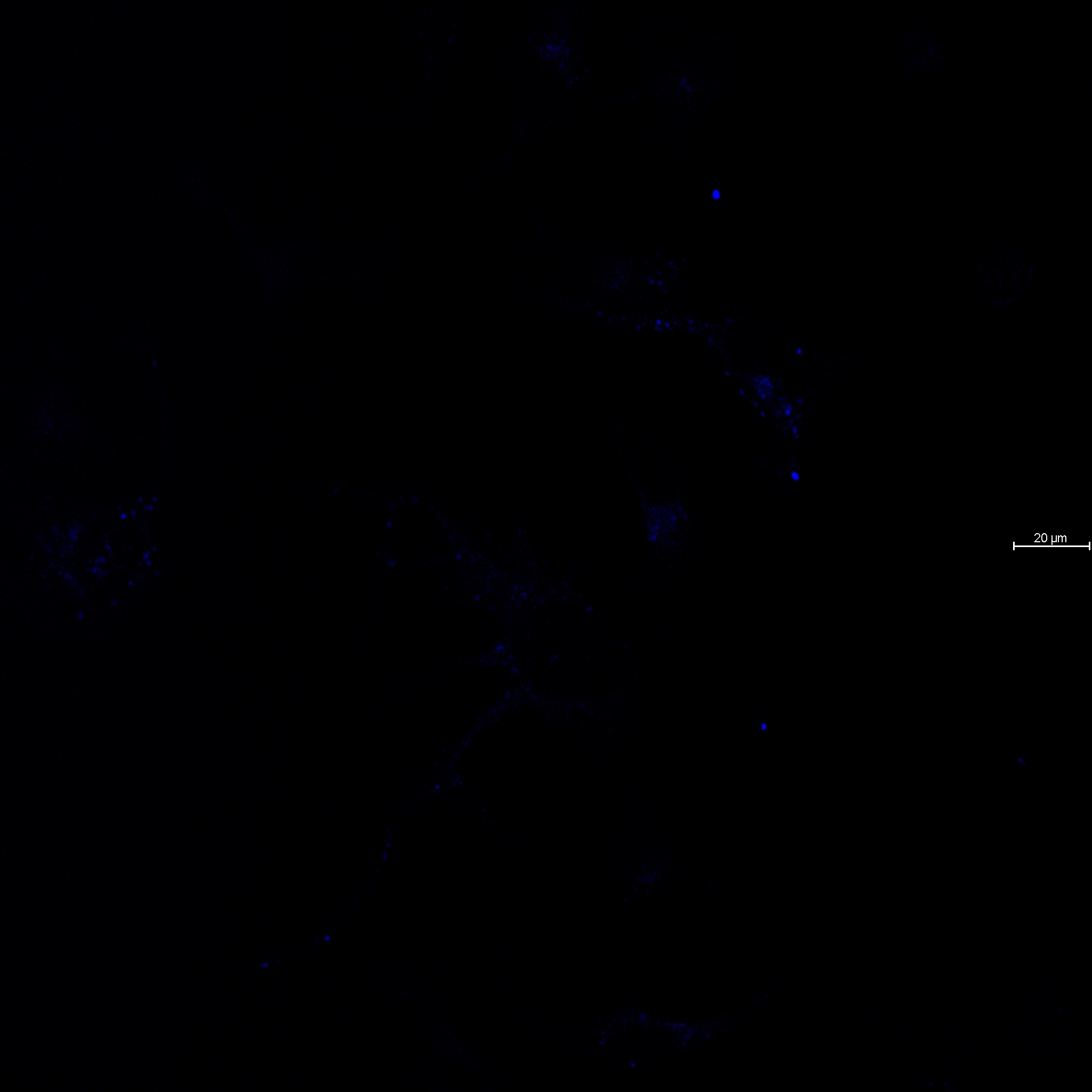

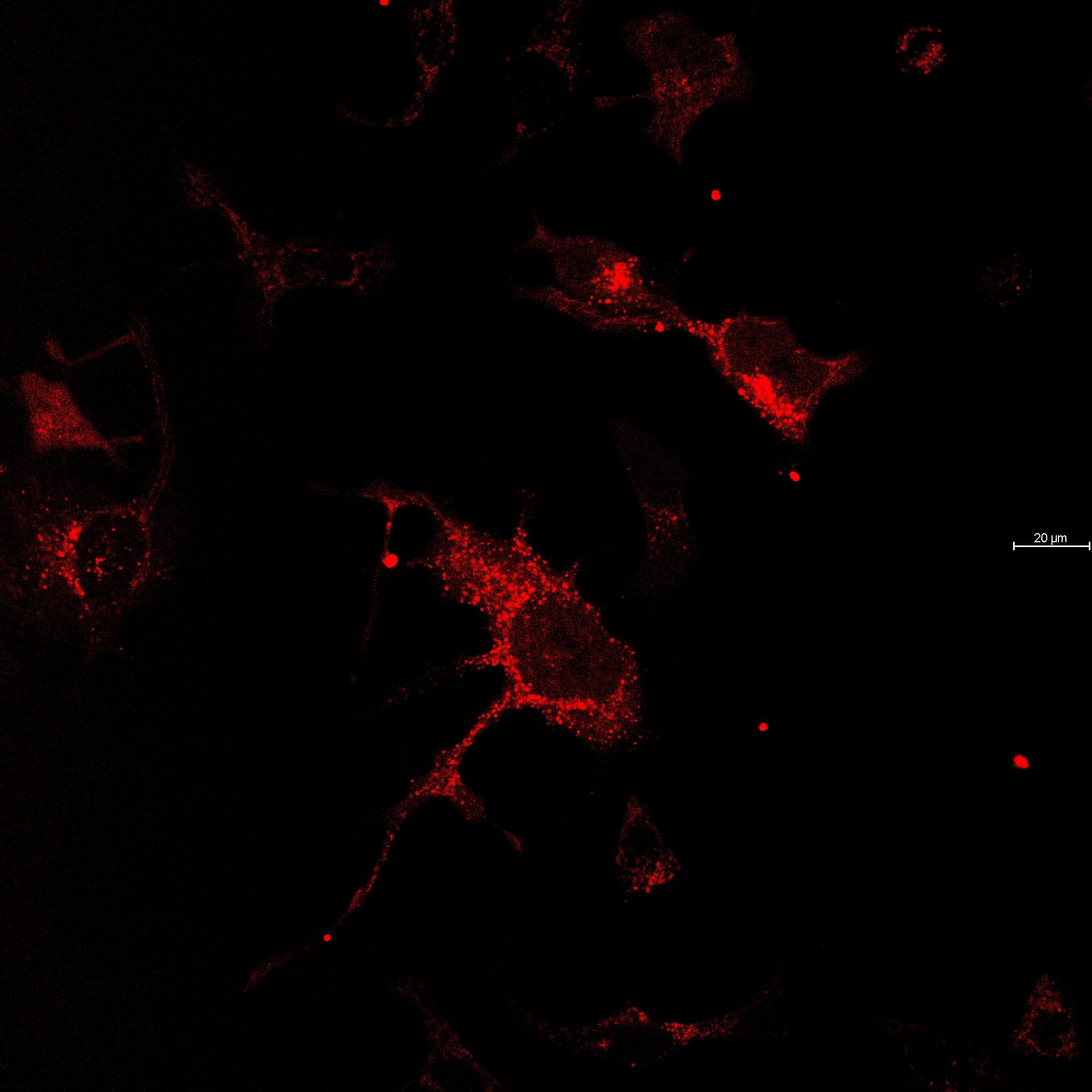


MCF-7

CD44 inhibitor

antibody


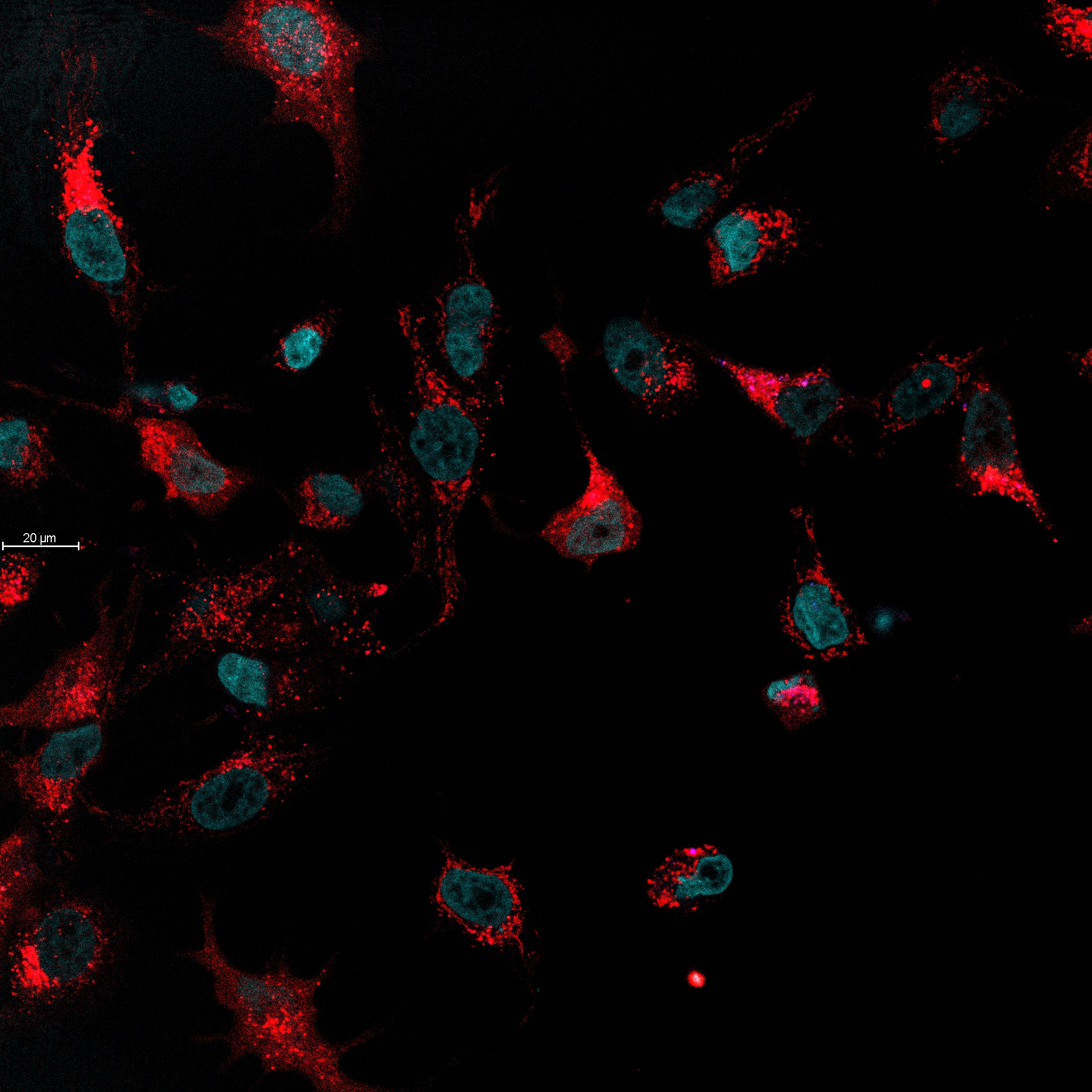

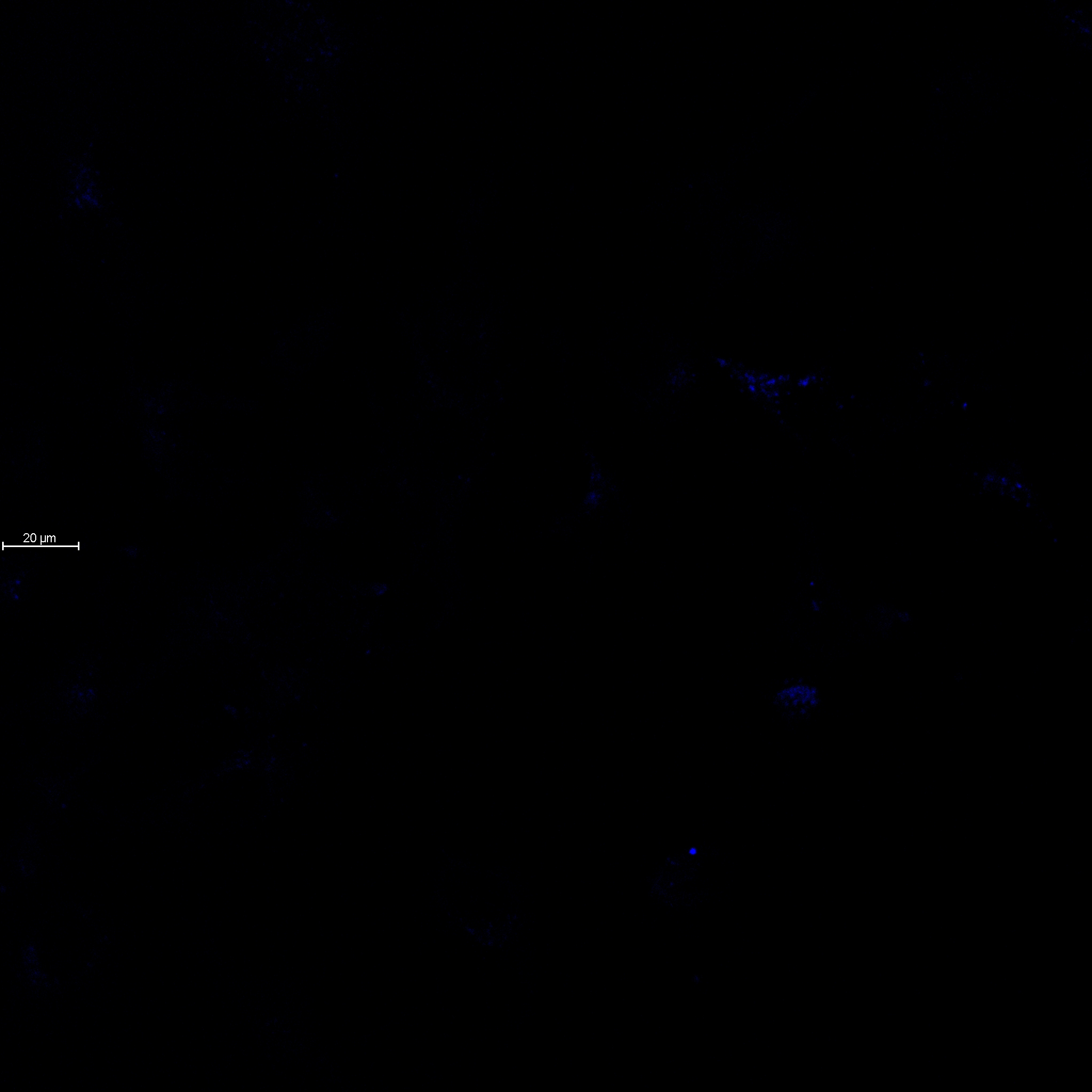

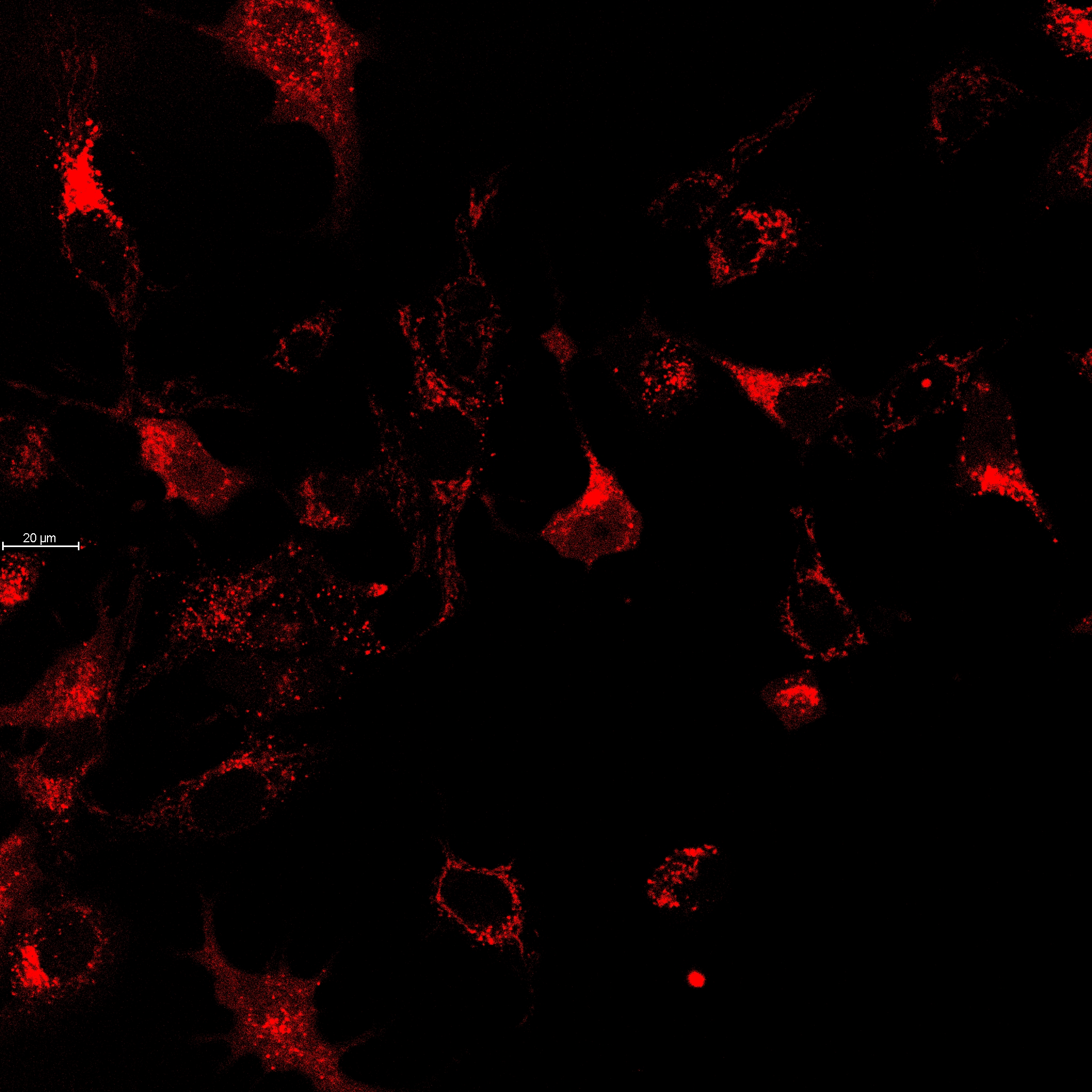


**Supplementary Figure S4.** Cellular internalization of **1** by DOK and MCF-7 cells

Confocal microscopy showing uptake of **1** in DOK and MCF-7 cells overlayed with pHrodo Red - a marker of endocytosis, and SYTO Deep Red Nucleic Acid Stain. Cells were treated with 2.5 μg/mL CD44 blocking antibody or isotype control antibody for 30 min, then cotreated with 20 μM 1 for 2 h, SYTO Deep Red Nucleic Acid Stain for 1 h, and 30 μg/mL pHrodo Red for 30 min in serum free medium. Cells were then washed twice with PBS and fresh serum free medium added immediately prior to imaging on Leica SP8 Confocal Microscope. Bar is 20 μm. Images were analysed using Leica Application Suite X version 3.5.5.19976, www.leica-microsystems.com.

**Supplementary Figure S5.** Cell viability of SCC-9 cells after 24 h exposure to compound **1** and its intermediates

Cells were treated with a range of concentrations of the polymer compounds for 24 h. The viability of the cells was assessed by Alamar Blue assay relative to a vehicle control (0.1% volume of MilliQ H_2_O or DMSO). The concentrations shown are in log scale. Values represent the mean ± SD of three independent experiments. IC_50_ values were 4.7 μM for **5**, 2 μM for **7**, and 19.7 μM for OA. Cell viability was 85% for 100 μM **1**, 115% for 100 μM **6** (HA-FA-Pg-3F), and 114% for 100 μM hyaluronic acid (HA8700). Graphs were plotted using GraphPad version 6.0c for Mac, www.graphpad.com.

**Supplementary Video 1.** Internalization of compound 1 by SCC-9 cells

3D confocal microscopy of compound 1 in SCC-9 cells. Immediately prior to imaging, a concentration of 0.1 µM fluorescein sodium salt was added to the medium to allow visualisation of the cell outline. Inverted colours. Bar is 20 µm. Images were analysed using Leica Application Suite X version 3.5.5.19976, www.leica-microsystems.com, 3D rendering was performed using Imaris version 9.5, www.imaris.oxinst.com.
